# Modulation of acoustic navigation behaviour by spatial learning in the echolocating bat *Rhinolophus ferrumequinum nippon*

**DOI:** 10.1038/s41598-020-67470-z

**Published:** 2020-07-01

**Authors:** Yasufumi Yamada, Yurina Mibe, Yuya Yamamoto, Kentaro Ito, Olga Heim, Shizuko Hiryu

**Affiliations:** 1 Program of Mathematical and Life Sciences, Hiroshima University, Higashi-Hiroshima, Hiroshima 739-0046 Japan; 2 Faculty of Life and Medical Sciences, Doshisha University, Kyotanabe, 610-0321 Japan; 3 Department of Frontier Bioscience, Hosei University, Koganei, 184-8584 Japan

**Keywords:** Animal behaviour, Auditory system, Psychophysics, Spatial memory

## Abstract

Using echolocation, bats receive acoustic information on their surroundings, which is assumed to help them sophisticatedly navigate complex environments. In this study, to understand spatial learning and acoustic sensing in bats, we investigated how flight and echolocation control changed in *Rhinolophus ferrumequinum nippon* as they learnt about their surroundings in an obstacle course that they flew through repeatedly. In these experiments, two testing environments (acoustically permeable and acoustically reflective) were prepared using chains and acrylic boards as obstacles to evaluate the interactive effects of spatial learning and flight environments. We found that bats reduced the meandering width of their flights and pulse emissions, and also seemed to reduce their shifts in pulse direction as they learnt more about their environments in both conditions. Throughout all our experiments, the bats with slower flight speeds tended to emit more pulses, which suggests that the number of pulse emissions reflects the echolocation tactics of each bat. The maximum flight speed was especially increased in the acoustically permeable condition, with frequent emissions of multiple pulses (≧triplets) in the early stages of flight, suggesting that bats adjust their flight plan based on how much of their surroundings they are able to sense in advance.

## Introduction

Echolocating bats form a mental representation of their surroundings using the ultrasonic signals they receive from active sensing^1^. They can navigate while successfully avoiding obstacles and capture prey with unpredictable obstacles in their surroundings (e.g. the flight patterns of nearby conspecifics)^2–5^. Using echolocation, bats acoustically scan to receive spatio-temporal information about their surroundings, which is assumed to help them navigate complex environments smoothly^6,7^. For example, echolocating bats may shift their acoustic gaze i.e. the direction of their ultrasound pulses in terms of their flight path, given that their acoustic field of view with one transmitter and two receivers is spatially limited^8^.For example, Japanese house bats (*Pipistrellus abramus*) emit pulses, not only towards their immediate target, but also towards their next intended target while chasing multiple prey^9^. This indicates that bats plan their future flight paths by controlling the beam direction of their pulses in advance in environments with unpredictable surroundings^10^.

**Figure 1:**
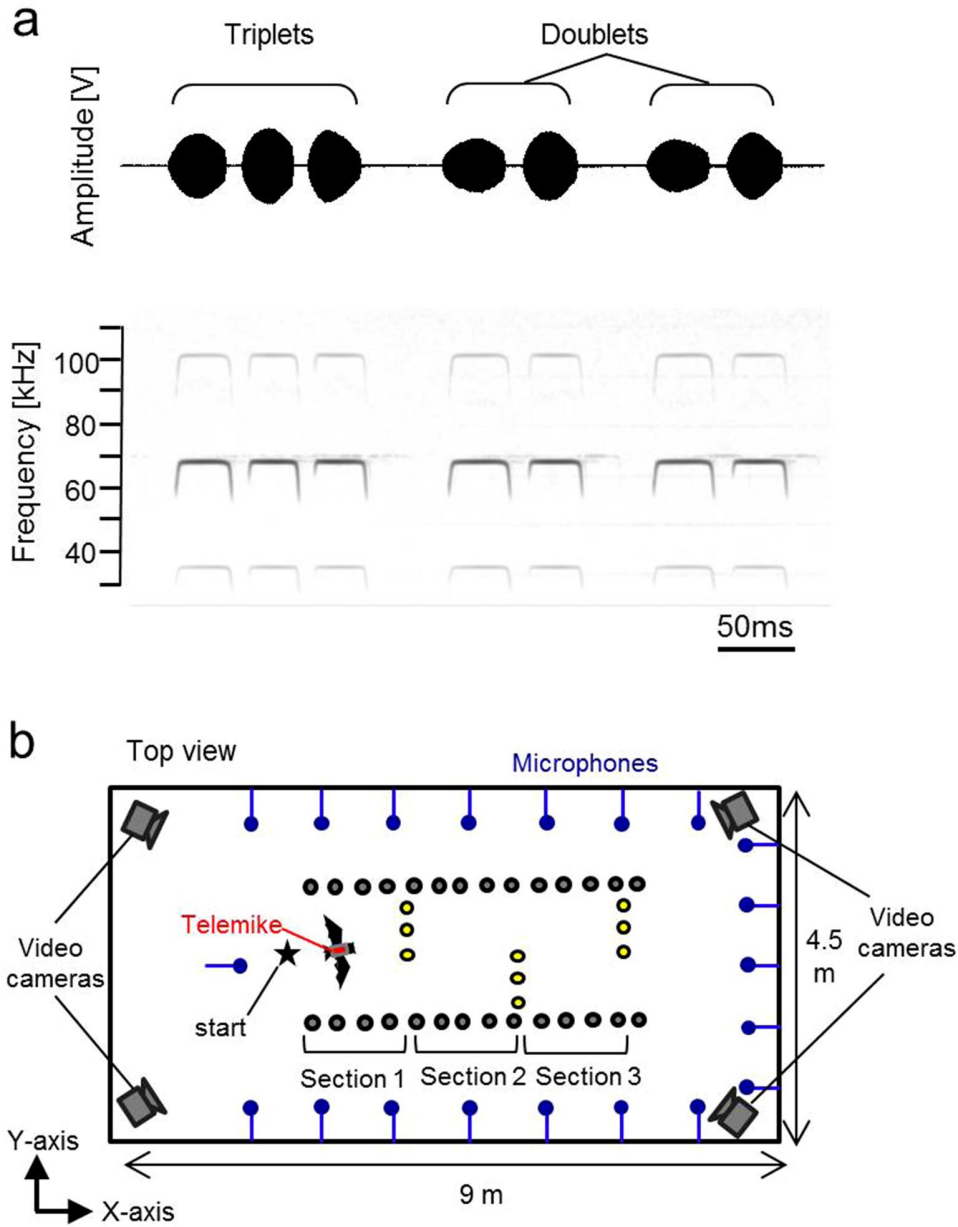
Echolocation calls of *R. ferrumequinum nippon* during repeated flights in the obstacle course in acoustically permeable and acoustically reflective wall conditions. (**a**) Amplitude pattern (top) and sonogram (bottom) of typical pulse emission sequences in *R. ferrumequinum nippon* during flight in the chamber. Sounds were recorded with the telemetry microphone mounted on the back of the bat. (**b**) Arrangement of the microphone array and obstacles in the flight chamber. To construct the acoustically permeable environment, three chains (yellow circles) were arranged at 22-cm intervals along the *y*-axis to form acoustically permeable walls. To create the acoustically reflective condition, we replaced the chain walls with acrylic boards. The three chain walls or acrylic boards were alternately arranged within an aisle, which was framed by chain walls (15-cm interval between chains, *x*-axis, grey circles), so that the bats were forced to follow an S-shaped flight pattern. For the behavioural analysis of the acoustic gaze, the flight state was separated into three sections, based on the location of each obstacle wall.

Bats are also widely known to flexibly control their pulse emission timings based on their surrounding environment and flight tasks^11–13^. During prey-capture flights, aerial-hawking bats decrease their inter-pulse interval as they approach their target prey to update the position of their target more frequently^14,15^. In addition, bats also often alternate between long and short interval pulse emissions. A set of multiple pulses emitted with short intervals were called strobe groups^15,16^. Previous studies for *Eptesicus fuscus* reported that these strobe groups increase when bats target an insect that moves unpredictably^12^ and when bats fly in narrow spaces surrounded by multiple obstacles^17–19^. These studies concluded that the temporal pattern of pulse emission timings is affected by the difficulty in predicting the spatial properties of flight environments^12,19^. Among strobe groups, shorter interval pulses in pairs and trios have been called doublets and triplets, respectively (Fig. 1a)^7,12^. The emission proportion of each doublet and triplet may or may not be influenced by environmental conditions and flight situations, but it has consistently been reported that *Eptesicus fuscus* seem to frequently repeat doublets during flights in high cluttered environments^7,12^. These findings suggest that doublets and triplets provide additional spatial resolution to locate nearby objects. Analysing the temporal patterns of pulse emission timings would enable us to find a behavioural solution for the problems faced by bats in cluttered environments.

While bats have a sensing strategy to guide them in cluttered environments as described before, they also have a more adaptive strategy that relies on spatial memory to help them navigate cluttered environments. For example, *Eptesicus fuscus* were shown to reduce their pulse repetition rate and rapidly stabilise their flight path to circuit the same area, based on their daily flight experiences in the same cluttered environment^2^. These adapted behaviours did not reset even when their starting flight position was changed. Flight path stabilisation was also shown in insect-capturing flights in cluttered environments, but the reduction of pulse emissions could not be observed, suggesting that reducing pulse emissions using spatial memory is difficult for bats in foraging flights that require more accurate navigation^18^. For one of the spatial learning strategies used during feeding flights, it was suggested that *Eptesicus fuscus* learn the relative location of feeding environments based on position of certain acoustic landmarks^20^. Thus, bats also have the ability to adapt their echolocation and flight behaviours by using spatial memory to reduce their sensing costs and to increase flight safety or certainty.

The studies mentioned above on *Eptesicus fuscus* suggest that changes in echolocation behaviours are influenced by two main factors: flight environment and spatial familiarity. These two factors are both affected by the prediction difficulty of flight environments. In places where acoustic permeability is poor, for example, when echolocating bats face an acoustically reflective wall such as a large panel, ultrasonic propagation is blocked by the wall; bats cannot detect the space behind it. If bats used their spatial memory while also sensing ahead as much as possible when planning their flight paths, such a reflective wall would make it difficult for bats to plan their flight path. Therefore, we hypothesise that differences in the degree of flight path modulation and echolocation would be present in scenarios in which acoustic permeability is either good or poor. If our hypothesis is rejected i.e. there are no behavioural differences between good or poor acoustic permeability conditions, it suggests that bats strongly compensate for the lack of information on their surrounding environment by using their accurate spatial memory, and not their sensing abilities. Thus, our aim is to understand the dependence balance between active sensing and spatial memory based on a behavioural comparison between good or poor acoustic permeability conditions. In particular, given that acoustic gaze is also a parameter that may be used to investigate the spatio-temporal attention of an animal^9,21^, we aim to examine how bats change the control of their spatio-temporal echolocation by focusing on the emission of multiple pulses, pulse directions, and flight paths.

To verify our hypothesis, obstacle courses of the same layout were constructed in a chamber with two different materials i.e. acoustically permeable walls and acoustically reflective walls, and then twelve repeated flights of *Rhinolophus ferrumequinum nippon* (*R. ferrumequinum nippon*) in the obstacle courses were measured by focusing on the flight path, the emission of multiple pulses and the pulse direction.

## Results

The obstacle layout was arranged to be a course which forced bats to fly with an S-shape flight path (Fig. 1b). With this layout, two different environmental conditions were prepared, one with an acoustically permeable wall and one with an acoustically reflective wall. Figure 1b shows an aerial view of the testing environment and the obstacle layout for the acoustically permeable wall condition. In the acoustically permeable wall condition, since all obstacle walls were constructed with an array of multiple plastic chains (diameter of 4 cm), bats would be able to detect the space in front of the permeable wall through the gaps in the chains. On the other hand, in the acoustically reflective condition, the three small chain walls (yellow circles in Fig. 1b) were replaced with acrylic boards [1 m (W) × 2 m (H)] so that the bats would not be able to detect the space behind the boards. Thus, two different conditions with the same obstacle layout were arranged to investigate behavioural differences based on the acoustical permeability of the flight environment.

Fourteen *R. ferrumequinum nippon* were divided into two groups so that each condition was tested with seven individual bats. Figure 1a shows representative echolocation pulses produced by *R. ferrumequinum nippon*. The spectrogram (bottom panel) shows that emission pulses are compound signals. Each signal consists of a constant frequency (CF) component and a second harmonic (CF_2_) of 68–70 kHz, which is the strongest component. This is accompanied by an initial, short, upward frequency modulated (FM) sweep and a terminal, short, downward FM sweep.

All bats used were naïve to both obstacle layouts. Therefore, the first flight in each condition can be regarded as an “unfamiliar space” flight. To examine how *R. ferrumequinum nippon* optimises flight behaviour and acoustic sensing as they become familiar with an obstacle environment, flights were repeated 12 times and measured for each individual bat, and then behavioural changes in the 1st and 12th flights were analysed. If bats coordinate flight relying on their spatial memory rather than acoustic sensing, there would be no remarkable differences in the learned flight behaviours between the acoustically permeable wall and reflective wall conditions. On the other hand, if it is necessary to detect a memorised landmark object for behavioural adaptation, reflective walls would prevent the detection of objects that are behind these walls. Therefore, the effect of spatial learning would be inhibited in the reflective wall condition. Based on these two alternative hypotheses, the flight speed, number of pulse emissions, and pulse directions were analysed for the 1st and 12th flights in each individual bat.

Overall, the statistical models that we describe in the Methods and Materials section (please see Supplemental Table S1 online for a detailed overview) fit the data well and explain more variance than the respective null models with R_marginal_^2^-values ranging between approximately 28% and 69% (please find further details in the Supplemental Table S2 online).

### Flight behaviour

**Figure 2:**
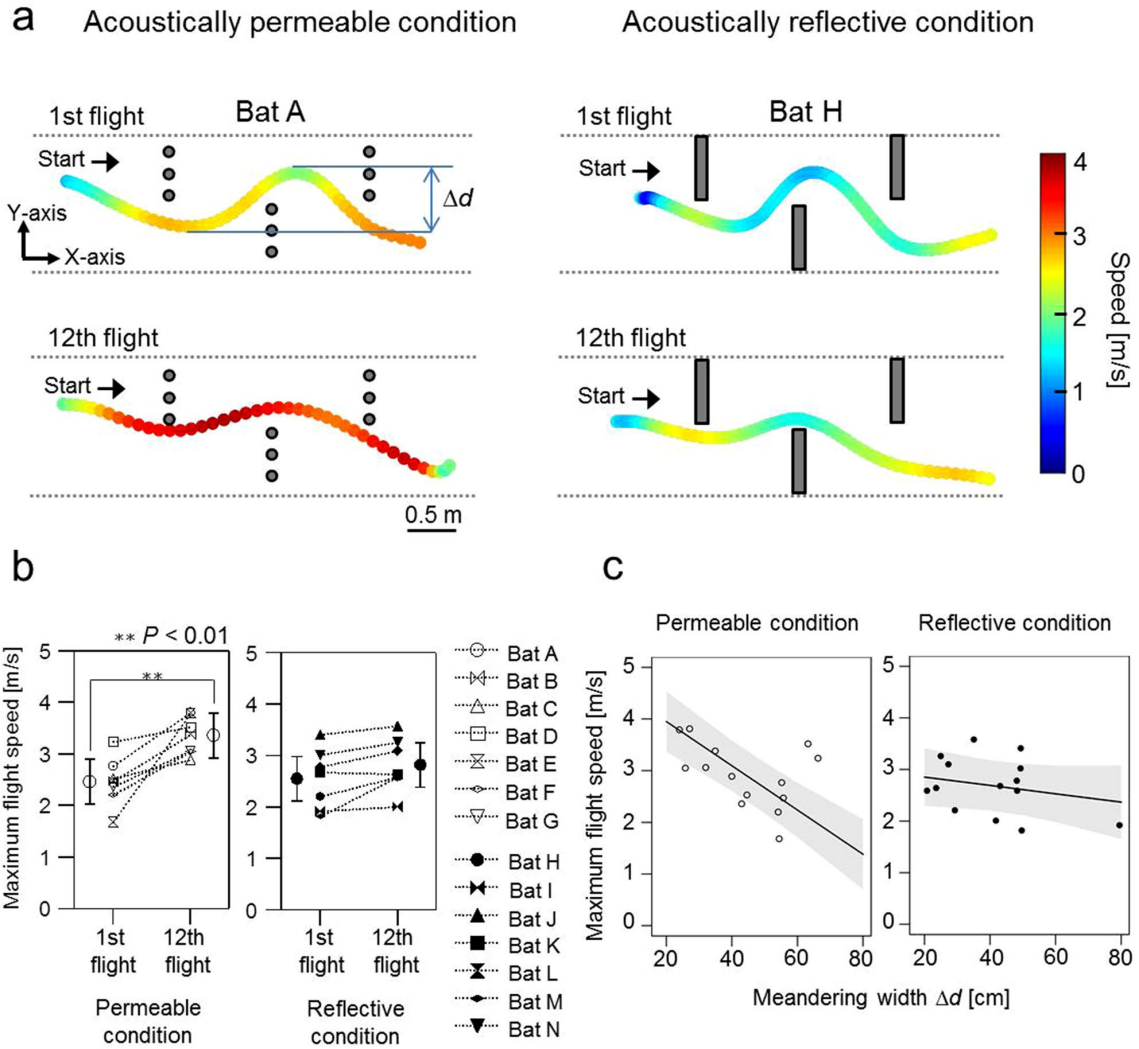
Movement behaviour of *R. ferrumequinum nippon* during repeated flights in the obstacle course in acoustically permeable and acoustically reflective wall conditions. (**a**) Exemplary flight paths and flight speeds between the 1st (top panel) and 12th (bottom panel) flight of bat A in the acoustically permeable wall condition and bat H in the acoustically reflective wall condition, respectively. (**b**) Relationship between the maximum flight speed, the flight number, and acoustical condition for all individuals’ data. (**c**) Relationship between the maximum flight speed, the meandering width Δ*d*, and the acoustic condition. Open and filled circles represent the raw data recorded in the acoustically permeable and reflective wall conditions, respectively. Black line and Grey shaded area in each panel indicate the predicted value and 95% confidence intervals, respectively.

Figures 2a shows examples of the flight path and speed of the 1st and 12th flights in the acoustically permeable wall (chains) and acoustically reflective wall (acrylic boards) conditions, respectively. All bats navigated the obstacle course without colliding with any obstacles (chain walls or acrylic boards) during the 12 consecutive flights. Moreover, the maximum flight speed significantly increased by about 1 m/s from the 1st to the 12th flight in the permeable wall (1st: mean = 2.5 m/s, SE = 0.2 m/s; 12th:, mean = 3.4 m/s, SE = 0.2 m/s; df = 29.2; *P* < 0.01; Fig. 2b, Supplemental Table S6 online) but not in the reflective wall condition (1st: mean = 2.6 m/s, SE = 0.2 m/s; 12th: mean = 2.8 m/s, SE = 0.2 m/s; df = 29.2; *P* = 0.4; Fig. 2b, Supplemental Table S6 online), leading to a significant interactive effect between flight number and acoustic condition (χ^2^ = 6.14, df = 1, *P* < 0.05, Supplemental Table S3 online). For all flights, Δ*d* was defined as the width of the meandering flight path (the peak-to-peak amplitude on the Y-axis of the first left turn to the following right turn of the S-shape flight path, see top panel of Fig. 2a). We found that bats in the acoustically permeable condition increased their flight speed from about 1.4–4 m/s while at the same time significantly decreasing Δ*d* (80–20 cm, Fig. 2c). This strong relationship between maximal flight speed and Δ*d* was not observed in the reflective wall condition (interactive effect: χ^2^ = 14.5, df = 1, *P* < 0.001, Supplemental Table S3 online). These results suggest that the flight speed control was affected by the presence of acoustic blind spots even after the bats became familiar with the environment. Thus, bats might depend on the availability of information about the space behind the immediate obstacle in order to modify flight speed during flight.

### Timing control of pulse emissions

Many previous studies have reported that bats of several species often emitted strobe groups with short time intervals during flight in cluttered environments^7,19^. In our analysis, strobe groups emitted with an inter-pulse interval of less than 40 ms is defined as a set of pulses^8^, and all pulses were classified into three types: multiple pulses (triplets or more), doublets, or a single pulse.

**Figure 3:**
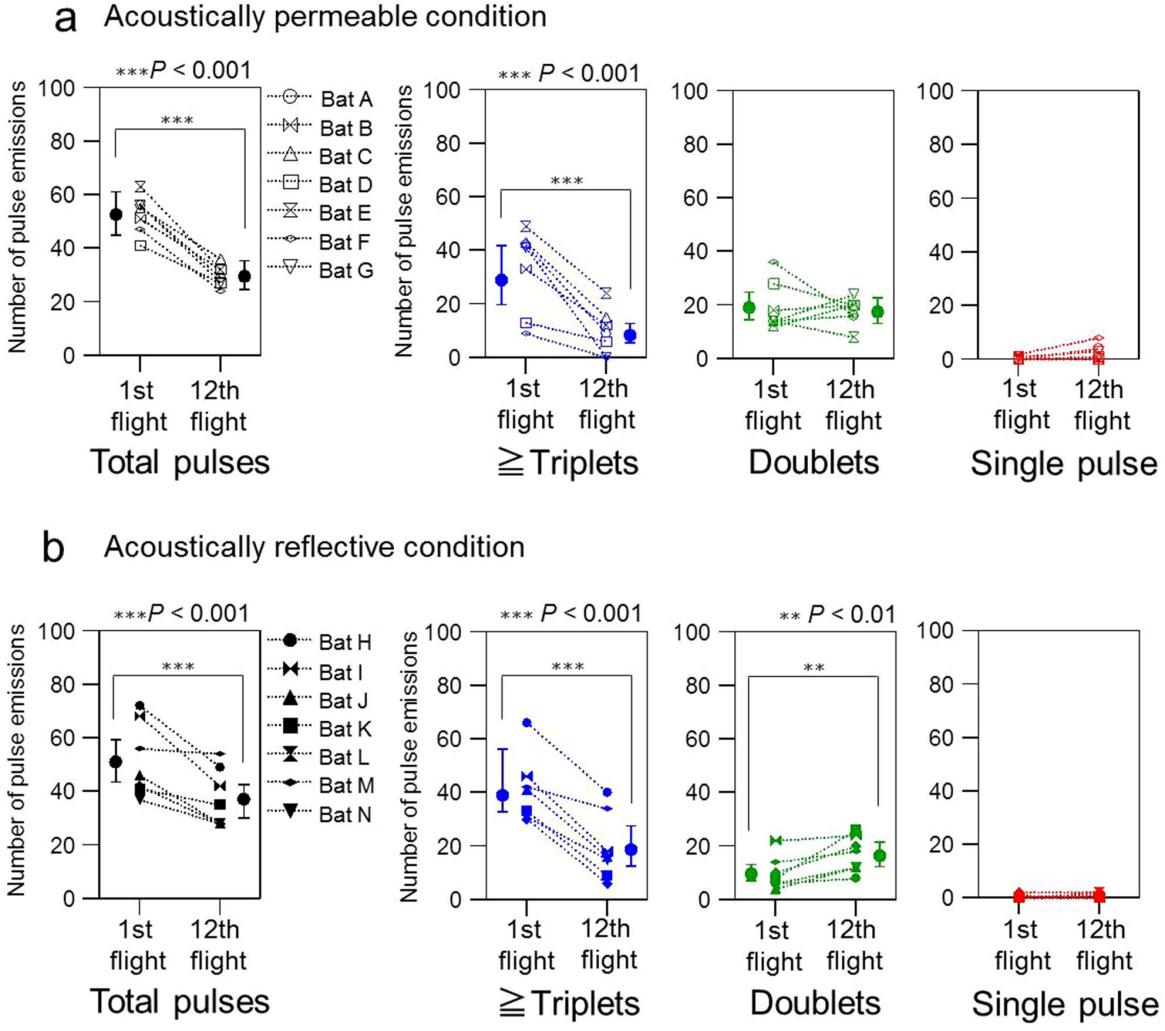
Relationship between the type and number of pulse emissions, the flight number, and acoustical conditions. All pulses (named *total pulses*, n = 7 bats per condition) were classified into three types: triplets or more pulses (n = 7 bats per condition, blue), doublets (n = 7 bats per condition, green), and single pulses (n = 7 bats per condition, red). Note that single pulses were not modelled due to insufficient data points. (**a**) Changes in echolocation behaviour between the 1st and 12th flight in the acoustically permeable condition. (**b**) Changes in echolocation behaviour between the 1st and 12th flight in the acoustically reflective wall condition.

Figure 3a,b show the changes in the number of pulses that bats emitted while flying through the obstacle course. After the bats became familiar with the space, the total number of pulse emissions dropped significantly from about 52 pulses in the 1st flight (SE = 1.1) to about 29 pulses in the 12th flight (SE = 1.1, *P* < 0.001, Supplemental Table S6 online) in the acoustically permeable condition. Such a drastic drop was also observed in the total number of emitted pulses in the reflective condition (1st: mean = 51.9, SE = 1.1; 12th: mean = 37.0, SE = 1.1; *P* < 0.001, Supplemental Table S6online). Although an interactive effect appeared to be an essential part of the model when compared to a model without interaction (Parametric bootstrap test: stat = 4.9, df = 1, *P* = 0.02), we didn’t find any interactive effects when comparing means of variable levels (Supplemental Table S4 online).The number of emitted multiple pulses (≧ triplets) dropped significantly too, by about 20 pulses in both acoustical conditions after the bats became familiar with the environment (Permeable: 1st: mean = 28.8, SE = 1.2; 12th: mean = 8.2, SE = 1.2; *P* < 0.001; Reflective: 1st: mean = 38.9, SE = 1.2; 12th: mean = 18.5, SE = 1.2; *P* < 0.001, Supplemental Table S6 online). However, this relationship between the number of emitted multiple pulses and the flight number shifted downwards in the acoustically permeable condition compared to the reflective condition by about 10 pulses, leading to a significant interactive effect (χ^2^ = 8.7, df = 1, *P* = 0.003, Supplemental Table S4 online).

In contrast to the results for the number of multiple pulses, we found that bats emitted a higher number of doublets in the acoustically reflective condition in the 12th flight compared to the 1st (1st: mean = 9.5, SE = 1.2; 12th: mean = 16.3, SE = 1.2; *P* = 0.002, Supplemental Table S6 online), while the number of emitted doublets remained at a relatively high level of about 17 (SE = 1.1) to 19 (SE = 1.2) pulses for the 1st and 12th flight, respectively, in the acoustically permeable condition (interactive effect: χ^2^ = 10.6, df = 1, *P* = 0.001, Supplemental Table S4 online).

More than 96% of total pulse emissions were emitted as doublets and triplets or multiple pulses in both the acoustically permeable (556/576 pulses) and reflective conditions (617/626 pulses), such that emissions of single pulses were rare and constituted less than 4% of the total pulse emissions. Due to this reason, we refrained from modelling the number of single pulses.

**Figure 4:**
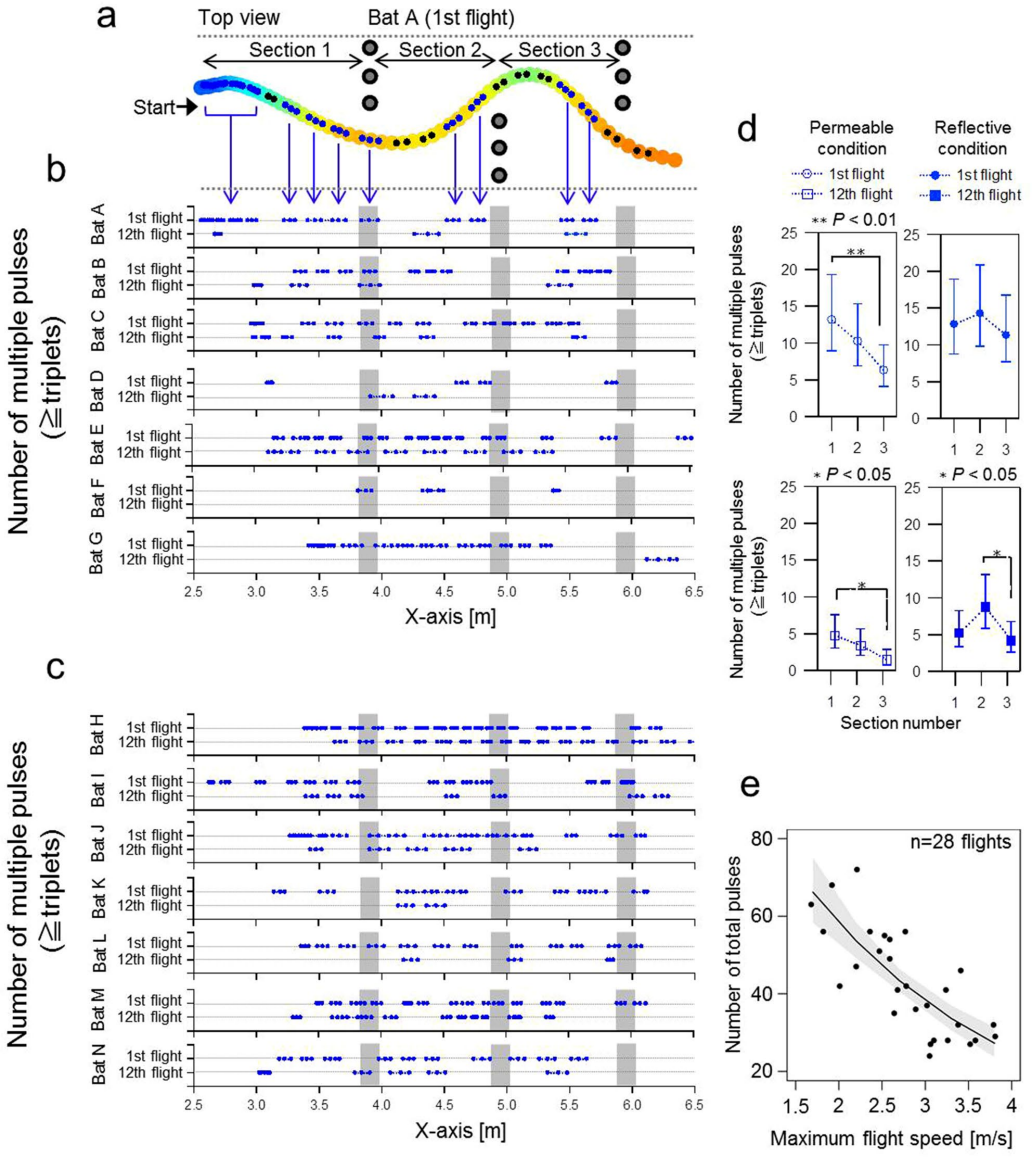
Emission of triplets across space and time. (**a**) Exemplary flight path of bat A as seen from above shows the spatial locations of triplet emissions as blue points. (**b**) Spatial locations of triplet emissions during the 1st and 12th flight of bats (A–G) passing the obstacle course in the acoustically permeable wall condition. (**c**) Spatial locations of triplet emissions during the 1st and 12th flight of bats (H–N) passing the obstacle course in the acoustically reflective wall condition. In both subplots (**b**) and (**c**), blue marker plots indicate the location of the emission of triplets, while grey shaded areas indicate the positions of the obstacle walls. (**d**) Change of the number of emitted triplets across obstacle course Sects. (1, 2 and 3) in the 1st and 12th flight and in the acoustically permeable (left panel) and reflective wall condition (right panel). Values for means and 95% confidence intervals were derived from the generalized linear mixed effect model. Closed and open plots in each panel indicate emissions during the 1st and 12th flight, respectively. (**e**) Relationship between the total number of emitted pulses and flight speed. Each plot represents the raw data recorded in the acoustically permeable and reflective wall conditions. Black line and Grey shaded area indicate the predicted value and 95% confidence intervals, respectively.

Figure 4a–c show the locations within the obstacle course where multiple pulses (≧ triplets) were emitted during the 1st and 12th flights of each individual. We observed that the locations of emitted multiple pulses (Fig. 4a) and their repetition frequency along the obstacle course varied among individuals (Fig. 4b,c). For example, in the acoustically permeable condition (Fig. 4b), the emission of triplets decreased after spatial learning took place for all bats. This was particularly true for bats F and G as they did not emit any triplets before passing the third wall. When comparing the number of multiple pulses emitted between different sections of the course (1, 2 and 3; Fig. 4d), we found a steady decrease of emitted pulses from the first to the last section in the 1st and the 12th flight within the acoustically permeable condition, with significant differences between the first and third section in both cases (1st flight—section 1: mean = 13.2, SE = 1.2; 1st flight—section 3: mean = 6.3, SE = 1.2; comparison: z-ratio = 4.3, *P* = 0.001; 12th flight—section 1: mean = 4.8, SE = 1.3; 12th flight—Section 3: mean = 1.5, SE = 1.4; comparison: z-ratio = 3.5, *P* = 0.02, Supplemental Table S7online). In contrast to the results in the acoustically permeable condition, we found that the number of multiple pulse emissions in the reflective condition remained at a relatively high level. We could observe only one significant decrease from about 9 multiple pulses in section 2 to about 4 multiple pulses in section 3 within the 12th flight (Fig. 4c; section 2: SE = 1.2; section 3: SE = 1.3; comparison: z-ratio = 3.4, *P* = 0.03, Supplemental Table S7 online).

Furthermore, we found that bats emitted considerably fewer pulses when flying at higher speeds compared to low flight speeds (β =  − 1.5, SE = 1.1, z-value =  − 7.504, *P* < 0.001; R_marginal_^2^ = 68.7%, R_conditional_^2^ = 73.9%; Fig. 4e, Supplemental Table S2 online), independently from the acoustic conditions. In particular, bats emitted about 66 pulses when flying at 1.7 m/s and reduced the number of pulses to 27 at a flight speed of 3.8 m/s.

### Acoustic gaze movements

During these experiments, sound recordings were also conducted using a 20-channel microphone array (see blue colours in Fig. 1b), which was arranged in the chamber, to analyse horizontal pulse direction. From the changes in the sound pressure levels of the pulses recorded in each channel of microphones, the direction of the maximum energy of the pulse was defined as pulse direction (see Fig. 7a); it was calculated in the same way as seen in our previous article^8^.

**Figure 5:**
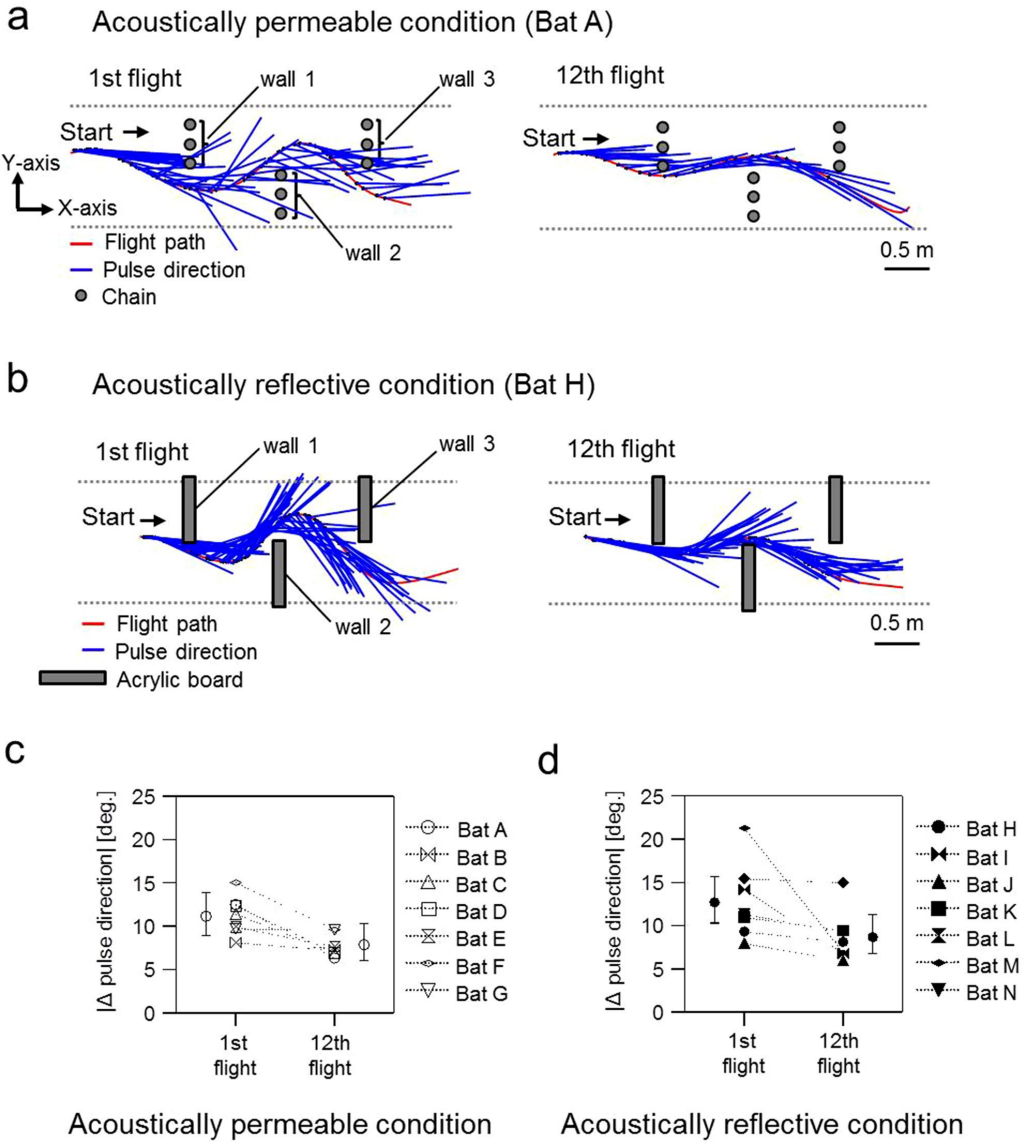
Directions of emitted pulses across space and time. (**a**, **b**) Aerial view of the 1st (left panel) and 12th (right panel) flight of bats (A and H). Flight path (red line) and the pulse directions (blue line) from the echolocation calls of flying bats in the obstacle course have been indicated. (**c,** **d**) Changes in average ∆pulse direction between the 1st and 12th flight in the acoustically permeable (**c**) and reflective (**d**) wall conditions. ∆pulse direction represents the change in the pulse direction between successive pulses (see Fig. 7b). Values for means and 95% confidence intervals were derived from the respective generalized linear mixed effect model.

The representative pulse direction changes are shown in Fig. 5a,b. In the acoustically permeable condition, bats tended to have more pronounced shifts in pulse direction relative to their flight direction in the 1st flight when compared to their 12th flight. On the other hand, in the acoustically reflective condition, bats tended to direct their pulse towards the flight direction during both the 1st and 12th flights. In order to describe these behavioural changes statistically, we calculated the Δpulse direction, which is the absolute amount of change in pulse direction between successive pulses (Fig. 7b), for all individuals. Although we found that the explanatory variable of the flight number explained a significant part of variance in the Δpulse direction (Type II Wald χ^2^-test, χ^2^ = 9.1, df = 1, *P* = 0.0026, Supplemental Table S1 online), post-hoc tests were inconclusive about whether the drop in the Δpulse direction between the 1st and 12th flight within both acoustic conditions was significant or not (χ^2^-test from package phia: permeable (1st vs. 12th): df = 1, χ^2^ = 3.94, *P* = 0.094; reflective (1st vs. 12th): df = 1, χ^2^ = 5.17, *P* = 0.046; contrasts from lsmeans: permeable (1st vs. 12th): z-ratio = 1.98, *P* = 0.28; reflective (1st vs. 12th): z-ratio = 2.27, *P* = 0.14; both test conducted with Bonferroni-adjustment for multiple comparisons, Supplemental Table S8 online).

**Figure 6:**
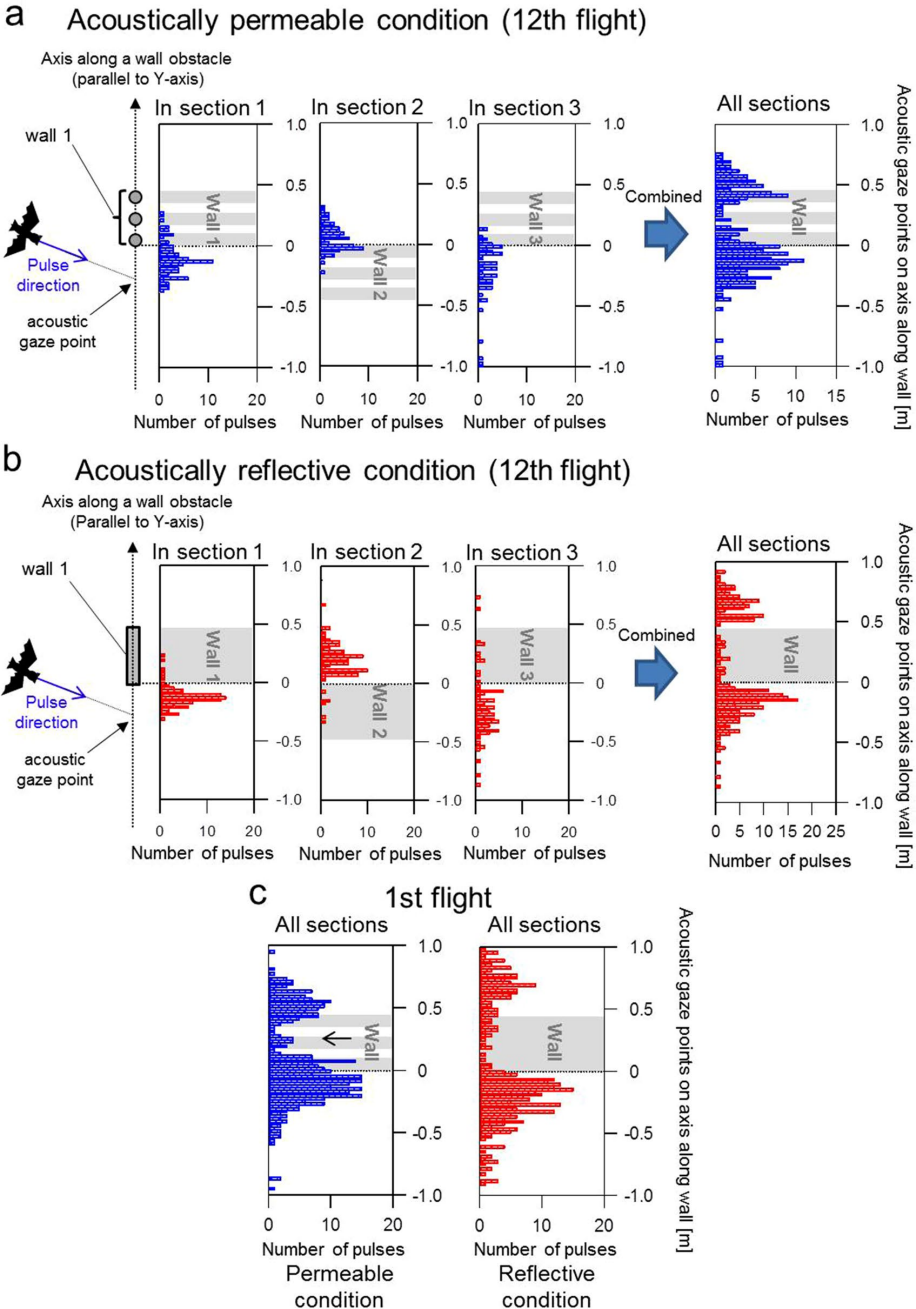
Comparison of the acoustic gaze dynamics of bats between the acoustically permeable and acoustically reflective wall conditions while flying through the obstacle course. (**a**, **b**) Histograms of the acoustic gaze points combined for all bats during the 12th flight in the acoustically permeable (**a**) and reflective (**b**) wall conditions. Note that the cross point of the pulse direction and the axis along an immediate obstacle wall was defined as the acoustic gaze point. The grey area indicates the immediate obstacle wall. Histograms for acoustic gaze points relative to the first, second, and third obstacle walls were made by separating the flight state into three sections (see Fig. 1b). An integrated histogram for all three sections in each condition is indicated in the upper-right and bottom-right panels, respectively. Note that the histogram was integrated with the vertical axis offset so that the wall locations of all three obstacles would be aligned. (**c**) Integrated histogram for all three sections during the 1st flight. The left and right-side panels indicate the acoustically permeable and reflective wall conditions, respectively.

If bats proactively paid attention to the space farthest away from them for flight path planning, their pulse direction in the acoustically permeable condition would more often be directed towards the inside of the wall they face, to detect the space behind the wall more precisely. In contrast, such pulse emissions might disappear in the reflective condition due to the undetectable space behind the reflective wall. In order to compare the emission proportion of pulses that are directed towards the inside of walls between the acoustically permeable and reflective conditions, the acoustic gaze point was defined as the point at which the line extending from the pulse direction intersects the axis along the closest wall (the schematic illustrations in Figs. 6a,b). The distribution of acoustic gaze points relative to the closest wall were separately analysed for each section of the flight state (see Fig. 1b) for all individuals and then summarised with histograms, as shown in Fig. 6a,b. The right-side panels in Figs. 6a,b also summarise the distribution patterns of the acoustic gaze points with all the data and flight sections during the 12th flight in each acoustically permeable and reflective condition. The emission rate of acoustic gaze points inside the walls with respect to the total number of pulses was 23% (46/204 pulses) for the acoustically permeable condition and 8% (21/258 pulses) for reflective condition in the 12th flight, indicating that the emission rate within walls in permeable condition was three times higher than that in the reflective condition. Figures 6c shows a summary of the distribution of acoustic gaze points during the 1st flight. Additionally, in the 1st flight, the emission rate within walls in the permeable condition was three times higher than that in the reflective condition. (7% = 25/354 pulses for the permeable condition and 25% = 93/366 pulses for the reflective condition). Moreover, in the histogram of the 1st flight of the permeable condition, a specific little peak appeared in the middle of the chain walls (black arrow shown in Fig. 6c). Thus, the bats were thought to sense the individual chains as well as the space beyond the chain wall through which the pulses passed.

## Discussion

In this study, we investigated whether bats could learn to control their flight and echolocation in a space that could test acoustically permeable and acoustically reflective conditions. The results from our behavioural experiment quantitatively show that flight and echolocation become more efficient with successive flights in both conditions. One of the indicators of echolocation efficiency is the number of pulse emissions. After repeated flights, we observed that the bats were able to avoid obstacles in their environment using less pulse emissions in both the acoustically permeable and reflective conditions^2^.This condition-independent behaviour indicates that bats have a high capability to compensate for missing information in acoustically undetectable areas using their spatial memory.

Additionally, in our experiments, *R. ferrumequinum nippon* usually produced doublets or triplets in any flights, which has been shown in previous flight experiments conducted in high cluttered environments using *Eptesicus fuscus*^7, 12,16^ and *Hipposideros armiger terasensis*^22^. One of the previous studies mentioned suggests that the temporal control of pulse emissions helps bats process high density echoes or navigate unfamiliar spaces^15^. In particular, doublets are regarded as a useful technique for bats to improve the resolution of an uncertain target’s position^12^. It has also been suggested that the information obtained from doublets contributes to flight path planning^16^. In the present study, we found that the number of single pulse emissions was very small when compared with the number of emissions of either doublets or triplets (Fig. 3). This indicates that the bats depended primarily on sensing with the emission of doublets or more pulses when exploring the acoustically cluttered environment.

*Eptesicus fuscus* were found to determine their own stable flight path and decrease the repetition rate of sonar broadcasts to a stable level as they became familiar with a space that contained an array of obstacles^2^. Interestingly, we found that although the emission of triplets decreased significantly, the emission of doublets did not decrease from the 1st to the 12th flights. This suggests that doublets are necessary elements in spatial searches even after spatial learning has taken place. On the other hand, when blind spots remain, multiple pulses (≧triplets) are more necessary to navigate unknown spaces because the bats in this study had not significantly reduced their multiple pulse emissions as their flight progressed in the acoustically reflective condition, as shown in Fig. 4d. Figure 4d also shows the frequent emission of multiple pulses in section 1 of the acoustically permeable condition, which immediately followed the start of their flight. The bats then decreased the emission of multiple pulses as their flight progressed in the acoustically permeable condition. This suggests that bats use multiple pulses to proactively detect obstacles ahead of them and to obtain information that may be used in the future during the early stages of flight.

The emission location of multiple pulses, which were used less frequently after spatial learning had taken place, was different for each individual (Figs. 4b,c). This result suggests that the use of multiple pulses may reflect the echolocation tactics of each individual bat. Especially, two individuals (Bat F and G) completely replaced triplets to doublets as they became familiar with the environment. In the future, we will investigate how to completely replace multiple pulses by analysing every series of flights. Figure 4e shows that the bats that flew with slower maximum flight speeds tended to emit more pulses. We can see the individuality of each bat reflected in their flight speeds, and that more cautious individuals fly more slowly and perform more sensing. Such differences in the navigation behaviours of individuals were also suggested in the research of visually guided human drivers by comparing the eye gaze movements between expert and ordinary drivers^23^. Along with the analysis of tactics common to individuals, carefully looking at the individuality of such acoustic navigation is an important part of analysing complex behaviour in bats, i.e. obstacle avoidance, in which there are a wide range of tactics that can be used by individuals to avoid obstacles.

In the present study, the measurement of acoustic gaze allowed us to evaluate spatio-temporal changes in the attention of the bats so that we could investigate their decision-making process with respect to spatial perception. Our experiment demonstrated that *R. ferrumequinum nippon* change their control strategy of pulse direction based on the acoustical permeability of their surrounding environment. In both the acoustically permeable and reflective conditions, bats often seemed to alternately shift their pulse direction during flight when they explored the initially unfamiliar environment, and then they tended to reduce the frequency of these shifts to direct their pulse ahead of their flight direction when they became familiar with the environment (see Fig. 5). Moreover, analyses of acoustic gaze points also show the behavioural differences between the bats in the acoustically permeable and reflective conditions. In the acoustically permeable condition, the bats often seemed to focus their attention far beyond the obstacle walls by directing their acoustic gaze to the permeable walls. However, in the acoustically reflective condition, the bats avoided gazing at the reflective walls and instead focused only on the edges of the reflective walls, where new undetected space appears when the bats fly forwards. Thus, their acoustic gaze movements were effectively adjusted to detect their flight environment as far away as possible, depending on the acoustic condition they faced. Moreover, it is a possibility that bats also adjusted their flight speed depending on the detected range of space by the current or previous sensing. These behavioural comparisons of acoustically permeable and reflective conditions suggest that bats coordinate their flights by referring to the distant space confirmed by sensing, rather than completely relying on pre-stored memory.

It has been previously reported that bats shift their acoustic gaze from side-to-side on their first flights, especially in this bat species, in which the beam width of emitted sound is narrow^8^. Furthermore, a shifting of the pulse direction has already been reported in various species of bats when chasing prey^24^, searching for insects^9^, or exploring environments^25^. We believe that our analysis of the change in acoustic gaze before and after spatial learning has taken place is useful for better understanding bat sonar tactics. For discovering the essence of adaptive navigation strategies in every environment, it is necessary to change the obstacle layout of the flight chamber and to further investigate the relationship between the use of multiple pulses and acoustic gaze during spatial learning.

In this study, we found that the bats not only chose an effective path planning^22,26^ method and sensing strategy, but also modulated these appropriately through the spatial learning of their surroundings. The ability to adapt navigation to any environment is an interesting topic, not only from an ethological perspective, but also from an engineering perspective. In order to navigate a completely unknown space, the navigating agent is required to localise both the environment and their own coordinates within the environment from sensing information. This simultaneous localisation and mapping (SLAM) problem has been recently well-investigated in engineering research fields^27,28^ and many solutions to the SLAM problem have been extensively proposed through practical demonstrations in both indoor^29–32^ and outdoor experiments^33,34^. In these systems, vehicle position in the surrounding environment is probabilistically estimated through the integration of spatial maps with single or multiple sensory input information obtained from global navigation satellite systems^33^, odometry sensors^34^, sonar^32^, laser range finders^29,31,34^, or stereo vision^30^. Moreover, a simple path planning method with spatial maps has also been proposed based on tracking system nodes and topological maps from which multiple check points (nodes) are extracted to plan a route^35,36^. So far, these navigation technologies seem to develop toward to improve the navigation safety based on the spatial map. On the other hand, our experiments showed that bats seemed to apply the spatial memory for reducing the energetic cost in navigation (e.g. reducing the sensing repetition rate and the wasteful turn movements) rather than increasing safety margin. If we could make the model as following the decision making process employed by bats that incorporates spatial familiarity, it has the potential to become a highly intelligent navigation system when integrated with SLAM systems. We suggest that the observed behavioural modulation of the bats during spatial learning may provide useful insights as a bio-inspired engineering system to bridge the navigation between unknown and known spaces.

## Materials and methods

### Subjects

Fourteen (seven male and seven female) adult *R. ferrumequinum nippon* were used in our experiments. Their body length was approximately 6.0–8.0 cm and their mean body mass was 27 g ± 3 g SD. The bats were captured from natural caves in the Hyogo and Fukui Prefectures in Japan. We carefully captured sleeping bats by wearing soft knit gloves on both hands and brought them back to our laboratory in soft knit bags. All capturing and transportation activities started in the early morning and finished while bats slept quietly. The capturing activities in Hyogo were conducted on October 6th, 2015, and those in Fukui were conducted on April 21st, 2016, May 8th, 2017, and March 27th, 2018, respectively. After our experiments, each bat was released back into the captured cave within 1 year.

All bats were housed in a special colony room [4 m (L) × 3 m (W) × 2 m (H)] at Doshisha University in Kyoto, Japan. In this colony room, the temperature and humidity were automatically maintained at 22 °C and > 70%, respectively. In addition, the interior lighting was controlled to provide a constant 12 h light and 12 h dark day-night cycle. While they were in the colony room, the bats could fly freely and were given access to mealworms and water placed against the walls. All licences required for capturing and rearing bats were obtained in the same way as in previous studies^8^, and these activities were conducted correctly in accordance with Japanese law for animal experimentation. In addition, all experimental procedures complied with the Principles of Animal Care (publication no. 86-23, revised 1985) issued by the National Institute of Health in the USA. Our experimental procedures were also pre-approved by the Animal Experiment Committee of Doshisha University.

While the bats were held in captivity, the body mass and echolocation sound for each bat were measured to check their health condition every week. Moreover, it was also confirmed that our experiment did not affect the health condition of each bat, based on these same measurements.

### Experimental conditions

The experiments were conducted in a flight chamber [9 m (L) × 4.5 m (W) × 2.5 m (H)] where multiple obstacles were suspended from the ceiling. Figure 1b shows an aerial view of the testing environment that was used in this study. The experiments were conducted in a flight chamber [9 m (L) × 4.5 m (W) × 2.5 m (H)]. With reference to previous studies assessing the electrical response of the retina of microchiropteran bats in four species^37^, it could be assumed that *R. ferrumequinum nippon* are also unable to respond to long wavelength light. Therefore, the inside of the chamber was illuminated with red-filtered light (> 650 nm) during the experiment to ensure that light does not affect the behaviour of the bats. The flight chamber was constructed with steel plates to prevent the interference of external electromagnetic signals due to broadcasts from FM radio stations.

An obstacle environment was constructed using either acoustically permeable or acoustically reflective walls that were vertically suspended from the ceiling. Two permeable chain walls were constructed by hanging chains at 15-cm intervals to create an aisle within the chamber (grey circles in Fig. 1b). Three chains (4 cm diameter) were then hung at 22-cm intervals perpendicular to the aisle walls to create a small wall (yellow circles in Fig. 1b). Three of these small chain walls were alternately arranged along the aisle so that the bats were forced to follow an S-shaped flight pattern through the aisle. The intervals between the chains of the walls were narrower than the average wing length of the bats, which was ~ 25 cm, to ensure that the bats could not pass through any of the walls. However, the bats were able to sense through all the permeable walls.

For the acoustically reflective wall condition, we replaced the three small chain walls (yellow circles in Fig. 1b) with acrylic boards measuring 1 m × 2 m (W × H). The acrylic boards did not permit the bats to sense the space behind the boards. The inside edges of the acrylic boards and the chain walls were placed in the same positions for each condition.

Our study was conducted using naïve bats for both obstacle layout conditions. First, the 14 bats were divided into two groups of seven bats each. Each group was assigned to either the acoustically permeable or acoustically reflective wall condition. The individual flights of all the bats were recorded and evaluated in each condition.

Given that none of the bats had any training prior to the beginning of the experiment, the first flight of each bat was regarded as a flight in a completely unknown space. The experimenter carefully carried each bat to the starting position at the entrance to the aisle in the flight chamber, holding it in their hands to prevent the bat from echolocating before recording began. The experimenter then made the bat fly from the starting position, which was located 1.3 m behind the first small wall (star in Fig. 1b). After every flight, the experimenter recaptured the bat that landed on the frontal wall of chamber by using the large butterfly net (open mouth diameter size is 50 cm) and returned it to the starting position in the same way as when entering the chamber for the subsequent flight. While holding the bat with one hand, the experimenter carefully supplied water to the bat with plastic pipette after every third flight. The experiment for each individual bat was completed within 10–30 min. In this manner, the echolocation behaviours of the bats in both familiar and unfamiliar spaces could be compared.

### Video and sound recordings

All recording procedures were the same as those used in our previous study^8^. Digital high-speed video cameras (MotionPro X3; IDT Japan, Inc., Tokyo, Japan; 125 frames per second) were used to record the flight movement of the bats. In order to prevent blind spots, four video cameras were located in the corners of the flight chamber. Of the four video camera images, the two that clearly showed the bats’ movements were used for the analysis. The three-dimensional (3D) positions of the flying bats and obstacles were reconstructed by the direct linear transformation method using motion analysis software (DIPPMotionPro, ver. 2.2.1.0, Ditect Corp., Tokyo, Japan). Their flight speed was measured from the 3D flight trajectory, and the maximum flight speed was determined from the start of the flight to when the bat passed the third obstacle wall.

For recording ultrasonic vocalisations and echoes during echolocation flight, a custom-made miniature telemetry microphone^3,8,38^ was used. The telemetry microphone was equipped with a 1/8-inch omnidirectional condenser microphone (Knowles, Model FG-3629, Itasca, IL, USA) and an FM radio transmitter that enabled wireless recording using FM receivers. The total weight of the telemetry microphone was sufficiently light (approximately 0.6 g) compared to the body mass of the bats, owing to its use of a 1.5-V hearing aid battery (Sony, Type SR521SW, Tokyo, Japan). In the experiment, a telemetry microphone was attached to each bat's back with double-sided adhesive tape. The microphone was set to face forward and was positioned ~ 1 cm from the nose leaf of the bat, between the right and left pinnae. The transmitted radio signals from the telemetry microphone were received by an FM antenna (RadioShack Corporation, Model 15-1859, TX, USA) suspended from the ceiling, and then demodulated by a custom-made FM receiver (Dia-medical Corporation, DTT-1000, Tokyo, Japan) to recover the ultrasonic broadcasts of the bats. The recovered signal was band-pass filtered from 20 to 150 kHz (NF Corporation, Model 3,625, Yokohama, Japan), and stored on a PC after being digitised with a high-speed data acquisition card (Model NI PXIe-6358, 16-bit, *f*_s_ = 500 kHz; National Instruments, Tokyo, Japan). In this recording system, an on/off signal control switch was employed as a recording trigger so that the video and sound data could be synchronously recorded and stored on the PC.

To measure the horizontal pulse direction during echolocation flight, a 20-channel microphone array was arranged on the walls surrounding the flight chamber 1.2 m above the floor (Fig. 1b). Microphones were placed 1.1 m apart along the side walls of the flight chamber and 0.75 m apart along the back wall of the chamber. In addition to the surrounding 20-channel microphone array, three microphones were embedded in each acrylic board to appropriately measure pulse direction. We used the same kind of telemetry microphone for the array as the microphones that were mounted on each subject. All signals recorded by the microphone array system were digitised in the same manner as the telemetry microphone recordings. These recordings were also conducted synchronously with the telemetry microphone recordings.

### Sound analysis

The call parameters investigated in this study were pulse direction and inter-pulse interval (IPI). Pulse direction analysis was conducted using the recordings from the microphone array, whereas the IPI was measured from the telemetry microphone recordings. Custom MATLAB (MathWorks, Natick, MA, USA) routines were used to extract individual pulses from a spectrogram of both recordings. The spectrogram was constructed using a Hanning window, the sample size of which was 1,024 points, including 512 0-filled points, and with 98% of the points overlapping.

#### Microphone array recording

Using the 20-channel microphone array data, horizontal pulse direction was analysed using the approach described in our previous article^8^. First, the pulse arrival times in each channel of the microphone array were estimated based on the 3D flight position of the bats and the immediate time of the pulse emission as measured by the telemetry microphone. Using these estimated pulse arrival times, the pulses recorded in each channel were extracted with our custom MATLAB routine programme. Next, to measure changes in the sound pressure levels of the pulses in each channel, the maximum energy of the downward FM sweep in the second harmonic (tFM_2_) component was measured from the spectrograms^8^. The sound pressure level of the pulse measured from each channel was then corrected for sound propagation loss in the air. The sound spreading loss was corrected based on the path length between each bat’s position and the position of each microphone channel. The sound absorption loss was also corrected using absorption coefficients that were previously measured (2.4 dB/m at 65 kHz)^8^. In addition, sensitivity differences in each microphone channel were also calibrated by conducting pre-measurements using an ultrasonic loudspeaker. During this pre-measurement, the ultrasonic loudspeaker (PT-R7 III, Pioneer Corporation, Kanagawa, Japan) was set up 1 m in front of the microphone and presented 3-ms tone burst signals to the microphone. The sound pressure level and frequency of the presenting sound were set to 107 dB and 65 kHz, respectively. By measuring the sound pressure level obtained from each microphone under the same conditions, the sensitivity differences among the microphones could be calibrated.

**Figure 7:**
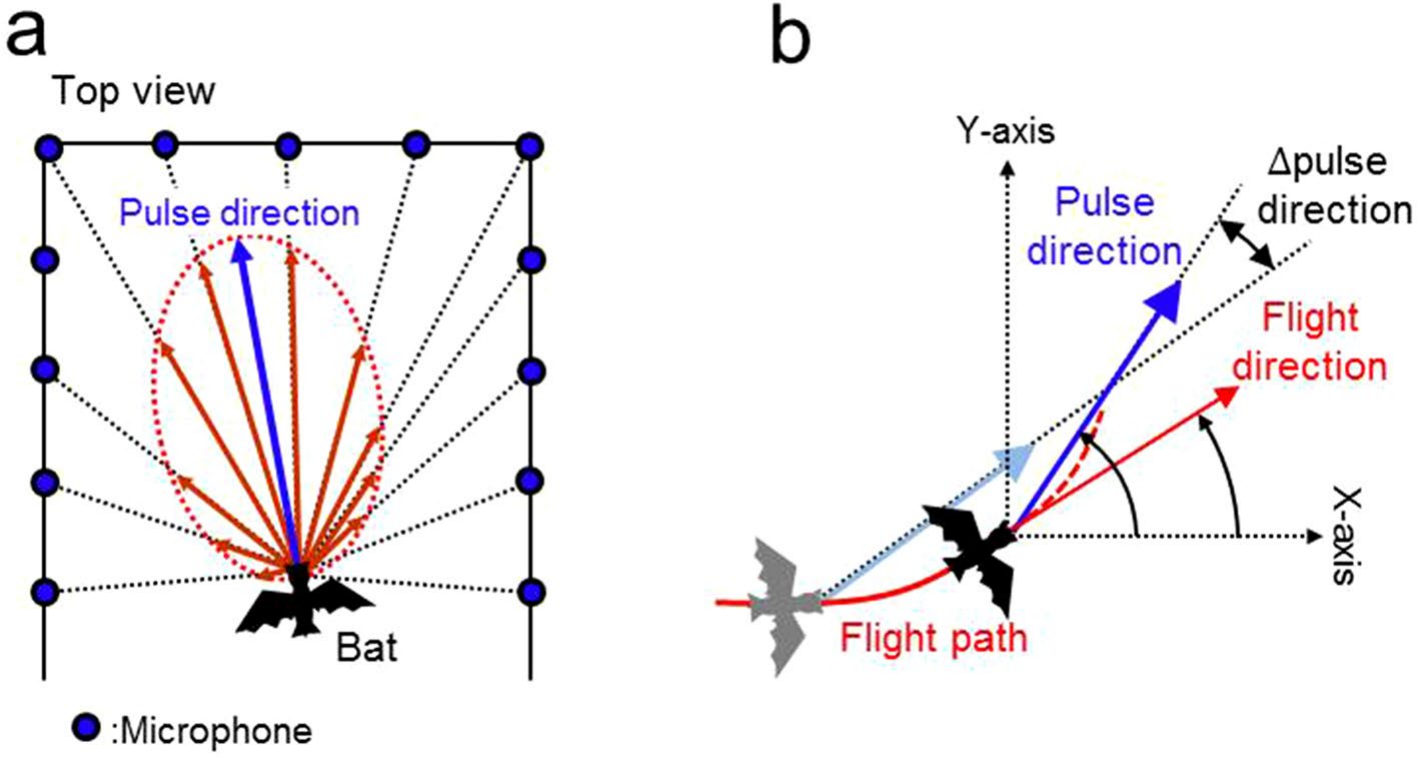
Methods used to derive pulse direction and Δpulse direction from the echolocation calls of flying bats. (**a**) Procedure of calculating the horizontal pulse direction using microphone array recordings. The pattern of pulse directivity (dashed red line) was reconstructed by integrating the sound pressure vectors across all microphones (red arrows). Then, the horizontal pulse direction was determined at the peak of the reconstructed pulse directivity pattern (blue arrow). (**b**) ∆pulse direction was defined as the angular differences between the previous and present pulse direction. Note that ∆pulse direction represents the absolute value.

For every pulse emission, the corrected sound pressure levels in each channel were converted to vectors, and Gaussian fitting was applied to the sound pressure vectors across all the microphones (red arrow, Fig. 7a). From this, the pulse directivity pattern was reconstructed. The horizontal pulse direction for each pulse was then determined from the energy maximum direction of the reconstructed pulse directivity pattern (blue arrow for horizontal pulse direction; Fig. 7a). The acoustic gaze angle was calculated as the angle from the flight direction to the pulse direction.

Measurement errors in pulse direction were evaluated in a previous article using an ultrasonic loudspeaker^39^. According to previous measurements, the directional errors of the pulses were less than 3°, ranging from 1 to 6 m on the frontal wall. Therefore, the obstacle course in this experiment was constructed in this accurate measurable area (2–5 m on the frontal wall).

Figure 7b shows the definitions of the horizontal angular components used in this study. The longitudinal and lateral axes in this chamber were defined as the X-axis (0°) and Y-axis (90°), respectively, in the horizontal plane. The ∆pulse direction was defined as the angular difference between the current pulse direction and the one immediately before.

#### Telemike recordings

Using the same MATLAB routines that were used for the microphone array recordings, the tFM_2_ of each pulse was extracted 25 dB from the maximum energy portion of the spectrogram. We used the tFM_2_ of each pulse to analyse emission timing and pulse direction. Pulse emission timing was defined as the beginning of the tFM_2_ of each pulse in the spectrogram (note that a time lag of approximately ten milliseconds occurs due to the elimination of the CF length, but there was no effect on the coordinates of the bats during flight in this study). The IPI was measured from the interval between the beginnings of the tFM_2_ of successive calls.

### Statistical analysis

To test our hypotheses, we modelled several measures of movement behaviour, such as flight speed and the number of emitted pulses, using generalised and linear mixed effect models (GLMM and LMM) within the framework of the statistical platform R (version 3.6.3)^40^. Due to the nature of our experimental design, we included bat-ID as a random effect into all our models (please find an overview of explained variance by random effects in Supplemental Table S9 online). Because our hypotheses are based on the expectation of behavioural changes occurring based on the interaction of acoustic conditions and the number of flights, we included the maximum number of interactive effects in all of our models.

In particular, we modelled flight speed as a function of acoustic condition in interaction with the flight number (1st vs. 12th) and the meandering width using LMM (function lmer, package lme4_1.1-21^41^), as the response variable is normally distributed. However, an initial data check showed a strong negative correlation between the meandering width and the flight number (Pearson’s correlation test, t =  − 4.1, df = 26, *p* value < 0.001, ρ =  − 0.63). As a result, we split this model into two, each containing either flight number or meandering width (Supplemental Table S1 online), and compared which model fit the data best using the Akaike Information Criterion corrected for a small sample size (AICc, function model.sel, package MuMIn_1.43.6^42^) (Supplemental Table S10).

We modelled the total number of emitted pulses, multiple and doublet pulses as a function of acoustic condition in interaction with the flight number. Furthermore, we modelled the total number of pulses as a function of flight speed in interaction with the acoustic condition, as well as a function of a three-way-interaction between acoustic condition, flight number, and the section of the obstacle course (1, 2 and 3). In addition, we modelled the absolute degree of the pulse direction (Δpulse direction) as a function of the acoustic condition in interaction with the flight number. For all of these models, we used GLMM (function glmer, package lme4_1.1-21^41^) and assumed a Poisson error distribution (log-link) as the above-mentioned response variables were either count data or data with integer numbers with a Poisson distribution (Supplemental Table S1 online).

In all cases, the fit of each model was checked by graphically examining the residuals of each model. As mentioned above, we routinely checked for overdispersion^44^. Furthermore, we tested whether the models explained more variance than the respective null models (that contained only the random effects) using a parametric bootstrap test with 1000 simulations (function PBmodcomp, package pbkrtest_0.4-7^45^^)^ (Supplemental Table S2 online). In addition, we calculated the overall variance that is explained by the model (function r.squaredGLMM, package MuMIn_1.43.6^42^). We determined whether individual variables within each model explained a significant portion of variance in each response by using a type II Wald-χ^2^-test (function Anova, package car_3.0-4^46^) (Supplemental Table S3, S4 and S5 online). Please find a detailed overview of effect sizes and standard errors in the Supplemental Tables S11 and S12 online. The values for means, 95% confidence intervals, between-level comparisons, degrees of freedom (df), and corresponding p-values were derived from respective models using the function lsmeans (package emmeans_1.4.3.01^47^) (Supplemental Table S6–S8 online). To correct the p-value for multiple comparisons, we used the Tukey-method. Please note that the function lsmeans does not support the calculation of degrees of freedom for GLMMs at the moment. Therefore, the value for df is not mentioned for GLMMs in the Results section.

### Ethical approval

The capturing and rearing of bats were conducted under licence and in compliance with the present Japanese law. Particularly, these activities complied with Fundamental Guidelines for Proper Conduct of Animal Experiment and Related Activities in Academic Research Institution (publication no. 71, published 2006) issued by the Ministry of Education, Culture, Sports, Science and Technology in the Japan. All experimental procedures complied with the Principles of Animal Care (Publication No. 86-23, revised 1985) issued by the National Institute of Health in the USA. Experimental procedures were also pre-approved by the Animal Experiment Committee of Doshisha University.

## Supplementary information

Supplementary information
