# Supplementary material for: Modulation of acoustic navigation behaviour by spatial learning in the echolocating bat Rhinolophus ferrumequinum nippon
Source: Sci Rep. 2020 Jul 1;10:10751. doi: 10.1038/s41598-020-67470-z (PMC7329871; doi:10.1038/s41598-020-67470-z)
Supplement: Supplementary file 1 — Supplementary information [file 41598_2020_67470_MOESM1_ESM.docx]

Title:
Modulation of acoustic navigation behaviour by spatial learning in the echolocating bat *Rhinolophus ferrumequinum nippon*

# Yasufumi Yamada, Yurina Mibe, Yuya Yamamoto, Kentaro Ito, Olga Heim, Shizuko Hiryu

**S1** Overview shows a list of response variables that were modelled using generalised (GLMM) and linear mixed effect models (LMM), respectively. GLMMs were modelled assuming a Poisson error distribution.

| **Response variable** | **Model no** | **Fixed and random factors** | **Model type** |
| --- | --- | --- | --- |
| **Maximum flight speed[m/s]** | **1** | $\text{F}\text{light }\text{n}\text{o}\text{ }\text{* }\text{C}\text{ond}\text{ }\text{+}\text{ }\text{(1\vert Cond/Bat)}$ | LMM |
| **Maximum flight speed [m/s]** | **2** | $\Delta\text{d}\text{ }\text{* }\text{C}\text{ond}\text{ }\text{+}\text{ }\text{(1\vert Cond/Bat)}$ | LMM |
| **Total number of pulses** | **3** | $\text{F}\text{light }\text{n}\text{o}\text{ }\text{* }\text{C}\text{ond}\text{ }\text{+}\text{ }\text{(1\vert Cond/Bat)}$ | GLMM |
| **Number of multiple pulses** | **4** | $\text{F}\text{light }\text{n}\text{o}\text{ }\text{* }\text{C}\text{ond}\text{ }\text{+}\text{ }\text{(1\vert Cond/Bat)}$ | GLMM |
| **Number of doublets** | **5** | $\text{F}\text{light }\text{n}\text{o}\text{ }\text{* }\text{C}\text{ond}\text{ }\text{+}\text{ }\text{(1\vert Cond/Bat)}$ | GLMM |
| **Number of multiple pulses** | **6** | $\text{F}\text{light }\text{n}\text{o}\text{ }\text{*}\text{ }\text{Sect}\text{ }\text{*}\text{ }\text{C}\text{ond}\text{ }\text{+}\text{ }\text{(1\vert Cond/Bat)}$ | GLMM |
| **Total number of pulses** | **7** | $\text{Speed}\text{ }\text{* }\text{C}\text{ond}\text{ }\text{+}\text{ }\text{(1\vert Cond/Bat)}$ | GLMM |
| **\|𝚫pulse direction\|** | **8** | $\text{F}\text{light }\text{n}\text{o}\text{ }\text{* }\text{C}\text{ond}\text{ }\text{+}\text{ }\text{(1\vert Cond/Bat)}$ | GLMM |

Abbreviations: Flight no = Flight number (1st flight vs. 12th flight), Cond = Acoustic condition (permeable vs. reflective), Bat = Individual bat ID, Sect = Flight chamber section (section 1 vs. section 2 vs. section 3; Fig. 1*b*), * = Asterisk indicates interaction between fixed effects

**S2** Summary of test statistics for comparisons between full models and their respective null models using parametric bootstrapping with 1000 simulations including adjusted R^2^-values for the full models.

| **Response variable** | **Model no** | **df** | ***P*** | **R^2^_mar._ [%]** | **R^2^_cond._ [%]** |
| --- | --- | --- | --- | --- | --- |
| **Maximum flight speed [m/s]** | **1** | 17.4 | ** | 33.0 | 71.6 |
| **Maximum flight speed [m/s]** | **2** | 15.6 | ** | 33.5 | 88.1 |
| **Total number of pulses** | **3** | 62.1 | ** | 55.6 | 78.0 |
| **Number of multiple pulses** | **4** | 153 | *** | 57.8 | 93.8 |
| **Number of doublets** | **5** | 16.8 | ** | 34.3 | 71.3 |
| **Number of multiple pulses** | **6** | 191 | ** | 58.0 | 83.8 |
| **Total number of pulses** | **7** | 54.0 | ** | 68.7 | 73.9 |
| **\|𝚫pulse direction\|** | **8** | 10.3 | ** | 27.7 | 27.7 |

Abbreviations: df = degree of freedom, R^2^_mar._ = marginal pseudo R^2^ indicates the explained model variation without random effects, R^2^_cond._ = conditional pseudo R^2^ indicates the explained model variation including random effects; P-values: *** = <0.001, ** = <0.01

**S3** Results from Wald χ^2^-tests (type II) made for linear mixed effect models of maximum flight speed as a function of either flight number (model 1) or meandering width *Δd* (model 2) in interaction with the acoustic condition. χ^2^ and p-values indicate which covariates (all 1 degree of freedom) affected the maximum flight speed. Interactions between covariates are indicated by colons. Interactive effects are shown in Figure 2*b* (model 1) and *c* (model 2).

| **Model no** | **Variables**  **Statistics** | **Flight no** | **Cond** | **Flight no : Cond** | ***Δd*** | ***Δd* : Cond** |
| --- | --- | --- | --- | --- | --- | --- |
| **1** | χ^2^ | 22.01 | 0.68 | **6.14** | NA | NA |
|  | *P* | n.s. | n.s. | ***** | NA | NA |
| **2** | χ^2^ | NA | 0.88 | NA | 30.53 | **14.50** |
|  | *P* | NA | n.s. | NA | *** | ******* |

Abbreviations: Flight no = Flight number (1st flight vs. 12th flight), Cond = Acoustic condition (permeable vs. reflective), $\Delta d$ = Meandering width (Fig. 2*a*), NA = not available –these variables were not included in the respective model; P-values: *** = <0.001, * = <0.05, n.s. = not significant

**S4** Results from Wald χ^2^-tests (type II). χ^2^ and p-values indicate which covariates (all 1 degree of freedom) affected the investigated response variables. Interactions between covariates are indicated by colons. All effects that are marked in bold font are presented as graphs in the main text.

| **Response variable** | **Model no** | **Variables**  **Statistics** | **Flight no** | **Cond** | **Flight no: Cond** | **Speed** | **Speed: Cond** |
| --- | --- | --- | --- | --- | --- | --- | --- |
| **Total number of pulses** | 3 | χ^2^ | 54.79 | 0.51 | **4.90** | NA | NA |
|  |  | *P* | *** | n.s. | ***** | NA | NA |
| **Number of multiple pulses** | 4 | χ^2^ | 123.40 | 2.68 | **8.66** | NA | NA |
|  |  | *P* | *** | n.s. | ****** | NA | NA |
| **Number of doublets** | 5 | χ^2^ | 2.95 | 3.25 | **10.56** | NA | NA |
|  |  | *P* | • | • | ****** | NA | NA |
| **Total number of pulses** | 7 | χ^2^ | NA | 0.01 | NA | **56.5** | 0.03 |
|  |  | *P* | NA | n.s. | NA | ******* | n.s. |
| **\|𝚫pulse direction\|** | 8 | χ^2^ | 9.09 | 1.02 | **0.01** | NA | NA |
|  |  | *P* | ** | n.s. | **n.s.** | NA | NA |

Abbreviations: Flight no = Flight number (1st flight vs. 12th flight), Cond = Acoustic condition (permeable vs. reflective), Speed = Maximum flight speed [m/s], NA = not available – these variables were not included in the respective model; P-values: *** = <0.001, ** = <0.01, * = <0.05, • = <0.1, n.s. = not significant

**S5** Results from a Wald χ^2^-test (type II) for the number of multiple pulses modelled as a function of flight number, acoustic condition and flight chamber section in a three-way-interaction. χ^2^ and p-values indicate which covariates (all 1 degree of freedom) affected the number of multiple pulses. Interactions between covariates are indicated by colons. The effect of the three-way-interaction is shown in Figure 4*d*.

| **Model no** | **Variables**  **Statistics** | **Fl no** | **Sect** | **Cond** | **Fl no : Sect** | **Fl no : Cond** | **Sect : Cond** | **Fl no : Sect : Cond** |
| --- | --- | --- | --- | --- | --- | --- | --- | --- |
| **6** | χ^2^ | 116.55 | 26.09 | 1.95 | 4.27 | 4.80 | **14.73** | 1.70 |
|  | *P* | *** | *** | n.s. | n.s. | * | ******* | n.s. |

Abbreviations: Fl no = Flight number (1st flight vs. 12th flight), Sect = Flight chamber section (section 1 vs. section 2 vs. section 3; Fig. 1*b*), Cond = Acoustic condition (permeable vs. reflective); P-values: *** = <0.001, * = <0.05, n.s. = not significant

**S6** List of posthoc comparisons between levels of variables within the model for maximum flight speed (Fig. 2*b*) and the models for pulse numbers (Fig. 3). Values for estimate and the *t*-ratio are taken from LMMs, while values for ratios and *z*-ratios are taken from GLMMs. Note that the values for Estimate/Ratio and standard error (SE) are given on the response scale. Comparisons that are presented in Figure 2*b* and Figure 3*a* and *b* are marked in bold font.

| **Response variable** | **Model no** | **Contrast** | | | | | **Estimate/Ratio** | **SE** | **df** | ***t*-/*z*-ratio** | ***P*** | |
| --- | --- | --- | --- | --- | --- | --- | --- | --- | --- | --- | --- | --- |
|  |  | **Fl no** | **Cond** |  | **Fl no** | **Cond** |  |  |  |  |  |  |
| **Maximum flight speed [m/s]** | **1** | **1** | **refl.** | **-** | **12** | **refl.** | **-0.28** | **0.18** | **12** | **-1.57** | **0.4** | **n.s.** |
|  |  | 1 | refl. | - | 1 | perm. | 0.08 | 0.30 | 29 | 0.28 | 1.0 | n.s. |
|  |  | 1 | refl. | - | 12 | perm. | -0.81 | 0.30 | 29 | -2.70 | 0.05 | • |
|  |  | 12 | refl. | - | 1 | perm. | 0.36 | 0.30 | 29 | 1.20 | 0.6 | n.s. |
|  |  | 12 | refl. | - | 12 | perm. | -0.53 | 0.30 | 29 | -1.78 | 0.3 | n.s. |
|  |  | **1** | **perm.** | **-** | **12** | **perm.** | **-0.89** | **0.18** | **12** | **-5.07** | **0.001** | ****** |
| **Total number of pulses** | **3** | **1** | **refl.** | **-** | **12** | **refl.** | **1.37** | **0.11** | **Inf** | **3.91** | **5.4E-04** | ******* |
|  |  | 1 | refl. | - | 1 | perm. | 0.97 | 0.11 | Inf | -0.27 | 1.0 | n.s. |
|  |  | 1 | refl. | - | 12 | perm. | 1.73 | 0.21 | Inf | 4.57 | 2.9E-05 | *** |
|  |  | 12 | refl. | - | 1 | perm. | 0.71 | 0.08 | Inf | -2.99 | 0.01 | * |
|  |  | 12 | refl. | - | 12 | perm. | 1.26 | 0.16 | Inf | 1.87 | 0.2 | n.s. |
|  |  | **1** | **perm.** | **-** | **12** | **perm.** | **1.78** | **0.15** | **Inf** | **6.67** | **1.6E-10** | ******* |
| **Number of multiple pulses** | **4** | **1** | **refl.** | **-** | **12** | **refl.** | **2.09** | **0.22** | **Inf** | **7.17** | **4.6E-12** | ******* |
|  |  | 1 | refl. | - | 1 | perm. | 1.35 | 0.36 | Inf | 1.12 | 0.7 | n.s. |
|  |  | 1 | refl. | - | 12 | perm. | 4.71 | 1.35 | Inf | 5.39 | 4.1E-07 | *** |
|  |  | 12 | refl. | - | 1 | perm. | 0.65 | 0.18 | Inf | -1.59 | 0.4 | n.s. |
|  |  | 12 | refl. | - | 12 | perm. | 2.25 | 0.66 | Inf | 2.76 | 0.03 | * |
|  |  | **1** | **perm.** | **-** | **12** | **perm.** | **3.48** | **0.48** | **Inf** | **8.98** | **2.7E-14** | ******* |
| **Number of doublets** | **5** | **1** | **refl.** | **-** | **12** | **refl.** | **0.58** | **0.09** | **Inf** | **-3.60** | **0.002** | ****** |
|  |  | 1 | refl. | - | 1 | perm. | 0.50 | 0.11 | Inf | -3.23 | 0.007 | ** |
|  |  | 1 | refl. | - | 12 | perm. | 0.55 | 0.12 | Inf | -2.78 | 0.03 | * |
|  |  | 12 | refl. | - | 1 | perm. | 0.86 | 0.17 | Inf | -0.74 | 0.9 | n.s. |
|  |  | 12 | refl. | - | 12 | perm. | 0.95 | 0.19 | Inf | -0.27 | 1.0 | n.s. |
|  |  | **1** | **perm.** | **-** | **12** | **perm.** | **1.10** | **0.14** | **Inf** | **0.75** | **0.9** | **n.s.** |

Abbreviations: Fl no = Flight number (1st flight vs. 12th flight), Cond = Acoustic condition, perm. = permeable wall condition, refl. = reflective wall condition; P-values: *** = <0.001, ** = <0.01, * = <0.05, • = <0.1, n.s. = not significant; df = degrees of freedom (df for GLMMs are not yet provided by the R-function and are therefore given as infinite (Inf))

**S7** List of posthoc comparisons between the single levels of the variables flight chamber section (Sect), flight number (Flight no) and the acoustic condition (Cond) that were used in a three-way-interaction to explain the number of multiple pulses (model 6, Fig. 4*d*). Comparisons that are presented in Figure 4*d* are marked in bold font.

| **Contrasts** | | | | | | | **ratio** | **SE** | ***z*-ratio** | ***P*** | |
| --- | --- | --- | --- | --- | --- | --- | --- | --- | --- | --- | --- |
| **Sect** | **Flight no** | **Cond** |  | **Sect** | **Flight no** | **Cond** |  |  |  |  |  |
| **1** | **1** | **refl.** | **-** | **2** | **1** | **refl.** | **0.90** | **0.13** | **-0.78** | **1.0** | **n.s.** |
| **1** | **1** | **refl.** | **-** | **3** | **1** | **refl.** | **1.13** | **0.17** | **0.82** | **1.0** | **n.s.** |
| 1 | 1 | refl. | - | 1 | 12 | refl. | 2.44 | 0.46 | 4.70 | 1.7E-04 | *** |
| 1 | 1 | refl. | - | 2 | 12 | refl. | 1.46 | 0.23 | 2.37 | 0.4 | n.s. |
| 1 | 1 | refl. | - | 3 | 12 | refl. | 3.06 | 0.63 | 5.43 | 3.6E-06 | *** |
| 1 | 1 | refl. | - | 1 | 1 | perm. | 0.97 | 0.27 | -0.10 | 1.0 | n.s. |
| 1 | 1 | refl. | - | 2 | 1 | perm. | 1.25 | 0.35 | 0.79 | 1.0 | n.s. |
| 1 | 1 | refl. | - | 3 | 1 | perm. | 2.02 | 0.60 | 2.39 | 0.4 | n.s. |
| 1 | 1 | refl. | - | 1 | 12 | perm. | 2.66 | 0.81 | 3.21 | 0.06 | • |
| 1 | 1 | refl. | - | 2 | 12 | perm. | 3.75 | 1.21 | 4.10 | 0.002 | ** |
| 1 | 1 | refl. | - | 3 | 12 | perm. | 8.44 | 3.27 | 5.51 | 2.3E-06 | *** |
| **2** | **1** | **refl.** | **-** | **3** | **1** | **refl.** | **1.26** | **0.18** | **1.60** | **0.9** | **n.s.** |
| 2 | 1 | refl. | - | 1 | 12 | refl. | 2.72 | 0.51 | 5.36 | 5.5E-06 | *** |
| 2 | 1 | refl. | - | 2 | 12 | refl. | 1.63 | 0.26 | 3.11 | 0.08 | • |
| 2 | 1 | refl. | - | 3 | 12 | refl. | 3.42 | 0.70 | 6.04 | 1.0E-07 | *** |
| 2 | 1 | refl. | - | 1 | 1 | perm. | 1.09 | 0.30 | 0.30 | 1.0 | n.s. |
| 2 | 1 | refl. | - | 2 | 1 | perm. | 1.39 | 0.39 | 1.19 | 1.0 | n.s. |
| 2 | 1 | refl. | - | 3 | 1 | perm. | 2.26 | 0.66 | 2.78 | 0.2 | n.s. |
| 2 | 1 | refl. | - | 1 | 12 | perm. | 2.97 | 0.90 | 3.59 | 0.02 | * |
| 2 | 1 | refl. | - | 2 | 12 | perm. | 4.18 | 1.34 | 4.46 | 5.0E-04 | *** |
| 2 | 1 | refl. | - | 3 | 12 | perm. | 9.42 | 3.63 | 5.82 | 4.0E-07 | *** |
| 3 | 1 | refl. | - | 1 | 12 | refl. | 2.15 | 0.42 | 3.97 | 0.004 | ** |
| 3 | 1 | refl. | - | 2 | 12 | refl. | 1.29 | 0.21 | 1.56 | 0.9 | n.s. |
| 3 | 1 | refl. | - | 3 | 12 | refl. | 2.71 | 0.57 | 4.76 | 1.2E-04 | *** |
| 3 | 1 | refl. | - | 1 | 1 | perm. | 0.86 | 0.24 | -0.54 | 1.0 | n.s. |
| 3 | 1 | refl. | - | 2 | 1 | perm. | 1.11 | 0.31 | 0.35 | 1.0 | n.s. |
| 3 | 1 | refl. | - | 3 | 1 | perm. | 1.79 | 0.53 | 1.96 | 0.7 | n.s. |
| 3 | 1 | refl. | - | 1 | 12 | perm. | 2.36 | 0.72 | 2.79 | 0.2 | n.s. |
| 3 | 1 | refl. | - | 2 | 12 | perm. | 3.32 | 1.08 | 3.70 | 0.01 | * |
| 3 | 1 | refl. | - | 3 | 12 | perm. | 7.46 | 2.90 | 5.17 | 1.5E-05 | *** |
| **1** | **12** | **refl.** | **-** | **2** | **12** | **refl.** | **0.60** | **0.12** | **-2.53** | **0.3** | **n.s.** |
| **1** | **12** | **refl.** | **-** | **3** | **12** | **refl.** | **1.26** | **0.30** | **0.96** | **1.0** | **n.s.** |
| 1 | 12 | refl. | - | 1 | 1 | perm. | 0.40 | 0.12 | -3.03 | 0.1 | n.s. |
| 1 | 12 | refl. | - | 2 | 1 | perm. | 0.51 | 0.16 | -2.17 | 0.6 | n.s. |
| 1 | 12 | refl. | - | 3 | 1 | perm. | 0.83 | 0.27 | -0.58 | 1.0 | n.s. |
| 1 | 12 | refl. | - | 1 | 12 | perm. | 1.09 | 0.36 | 0.27 | 1.0 | n.s. |
| 1 | 12 | refl. | - | 2 | 12 | perm. | 1.54 | 0.53 | 1.25 | 1.0 | n.s. |
| 1 | 12 | refl. | - | 3 | 12 | perm. | 3.46 | 1.41 | 3.06 | 0.09 | • |
| **2** | **12** | **refl.** | **-** | **3** | **12** | **refl.** | **2.10** | **0.46** | **3.40** | **0.03** | ***** |
| 2 | 12 | refl. | - | 1 | 1 | perm. | 0.67 | 0.19 | -1.42 | 1.0 | n.s. |
| 2 | 12 | refl. | - | 2 | 1 | perm. | 0.86 | 0.25 | -0.54 | 1.0 | n.s. |
| 2 | 12 | refl. | - | 3 | 1 | perm. | 1.39 | 0.42 | 1.08 | 1.0 | n.s. |

**Continuation of S7**

| **Contrasts** | | | | | | | **ratio** | **SE** | ***z*-ratio** | ***P*** | |
| --- | --- | --- | --- | --- | --- | --- | --- | --- | --- | --- | --- |
| **Sect** | **Flight no** | **Cond** |  | **Sect** | **Flight no** | **Cond** |  |  |  |  |  |
| 2 | 12 | refl. | - | 1 | 12 | perm. | 1.82 | 0.57 | 1.92 | 0.7 | n.s. |
| 2 | 12 | refl. | - | 2 | 12 | perm. | 2.57 | 0.85 | 2.86 | 0.2 | n.s. |
| 2 | 12 | refl. | - | 3 | 12 | perm. | 5.77 | 2.27 | 4.46 | 5.1E-04 | *** |
| 3 | 12 | refl. | - | 1 | 1 | perm. | 0.32 | 0.10 | -3.66 | 0.01 | * |
| 3 | 12 | refl. | - | 2 | 1 | perm. | 0.41 | 0.13 | -2.82 | 0.2 | n.s. |
| 3 | 12 | refl. | - | 3 | 1 | perm. | 0.66 | 0.22 | -1.26 | 1.0 | n.s. |
| 3 | 12 | refl. | - | 1 | 12 | perm. | 0.87 | 0.29 | -0.41 | 1.0 | n.s. |
| 3 | 12 | refl. | - | 2 | 12 | perm. | 1.22 | 0.43 | 0.57 | 1.0 | n.s. |
| 3 | 12 | refl. | - | 3 | 12 | perm. | 2.75 | 1.14 | 2.45 | 0.4 | n.s. |
| **1** | **1** | **perm.** | **-** | **2** | **1** | **perm.** | **1.28** | **0.19** | **1.69** | **0.9** | **n.s.** |
| **1** | **1** | **perm.** | **-** | **3** | **1** | **perm.** | **2.08** | **0.36** | **4.27** | **0.001** | ****** |
| 1 | 1 | perm. | - | 1 | 12 | perm. | 2.74 | 0.52 | 5.33 | 6.2E-06 | *** |
| 1 | 1 | perm. | - | 2 | 12 | perm. | 3.85 | 0.83 | 6.27 | 2.4E-08 | *** |
| 1 | 1 | perm. | - | 3 | 12 | perm. | 8.67 | 2.63 | 7.11 | 7.5E-11 | *** |
| **2** | **1** | **perm.** | **-** | **3** | **1** | **perm.** | **1.62** | **0.29** | **2.69** | **0.2** | **n.s.** |
| 2 | 1 | perm. | - | 1 | 12 | perm. | 2.13 | 0.42 | 3.87 | 0.006 | ** |
| 2 | 1 | perm. | - | 2 | 12 | perm. | 3.00 | 0.66 | 4.97 | 4.4E-05 | *** |
| 2 | 1 | perm. | - | 3 | 12 | perm. | 6.75 | 2.08 | 6.20 | 3.7E-08 | *** |
| 3 | 1 | perm. | - | 1 | 12 | perm. | 1.32 | 0.28 | 1.28 | 1.0 | n.s. |
| 3 | 1 | perm. | - | 2 | 12 | perm. | 1.85 | 0.44 | 2.59 | 0.3 | n.s. |
| 3 | 1 | perm. | - | 3 | 12 | perm. | 4.17 | 1.33 | 4.46 | 5.1E-04 | *** |
| **1** | **12** | **perm.** | **-** | **2** | **12** | **perm.** | **1.41** | **0.35** | **1.36** | **1.0** | **n.s.** |
| **1** | **12** | **perm.** | **-** | **3** | **12** | **perm.** | **3.17** | **1.04** | **3.50** | **0.02** | ***** |
| **2** | **12** | **perm.** | **-** | **3** | **12** | **perm.** | **2.25** | **0.78** | **2.35** | **0.4** | **n.s.** |

Abbreviations: Flight no = Flight number (1st flight vs. 12th flight), Sect = Flight chamber section (1, 2, 3), Cond = Acoustic condition, perm. = permeable wall condition, refl. = reflective wall condition; P-values: *** = <0.001, ** = <0.01, * = <0.05, • = <0.1, n.s. = not significant

**S8** List of posthoc comparisons between single levels of variables explaining the absolute angle of the pulse direction (Fig. 5*c* and *d*). Here we also present the different results given by the emmeans and phia package, respectively, while using the same adjustment method (Bonferroni) for multiple comparisons. Comparisons that are presented in Figure 5*c* and *d* are marked in bold font.

| **Package** | **Method** | **Flight no** | **Cond** |  | **Flight no** | **Cond** | **Ratio** | **SE** | ***z*-ratio** | ***P*** | |
| --- | --- | --- | --- | --- | --- | --- | --- | --- | --- | --- | --- |
| emmeans | **Tukey** | **1** | **refl.** | **-** | **12** | **refl.** | **1.46** | **0.24** | **2.27** | **0.1** | **n.s.** |
|  |  | 1 | refl. | - | 1 | perm. | 1.14 | 0.18 | 0.85 | 0.8 | n.s. |
|  |  | 1 | refl. | - | 12 | perm. | 1.62 | 0.28 | 2.81 | 0.03 | * |
|  |  | 12 | refl. | - | 1 | perm. | 0.78 | 0.13 | -1.44 | 0.5 | n.s. |
|  |  | 12 | refl. | - | 12 | perm. | 1.11 | 0.21 | 0.56 | 0.9 | n.s. |
|  |  | **1** | **perm.** | **-** | **12** | **perm.** | **1.42** | **0.25** | **1.98** | **0.2** | **n.s.** |
|  | Bon | 1 | refl. | - | 12 | refl. | 1.46 | 0.24 | 2.27 | 0.1 | n.s. |
|  |  | 1 | refl. | - | 1 | perm. | 1.14 | 0.18 | 0.85 | 1 | n.s. |
|  |  | 1 | refl. | - | 12 | perm. | 1.62 | 0.28 | 2.81 | 0.03 | * |
|  |  | 12 | refl. | - | 1 | perm. | 0.78 | 0.13 | -1.44 | 0.9 | n.s. |
|  |  | 12 | refl. | - | 12 | perm. | 1.11 | 0.21 | 0.56 | 1 | n.s. |
|  |  | 1 | perm. | - | 12 | perm. | 1.42 | 0.25 | 1.98 | 0.3 | n.s. |
|  |  |  |  |  |  |  | **Value** |  | **χ^2^** |  |  |
| phia | Bon | 1 | refl. | - | 12 | refl. | 1.46 |  | 5.16 | 0.046 | * |
|  |  | 1 | perm. | - | 12 | perm. | 1.42 |  | 3.94 | 0.09 | • |

Abbreviations: Bon = Bonferroni, Flight no = Flight number (1st flight vs. 12th flight), Cond = Acoustic condition, perm. = permeable wall condition, refl. = reflective wall condition; P-values: *** = <0.001, ** = <0.01, * = <0.05, • = <0.1, n.s. = not significant

**S9** Summary of random effect variances (Var.) and standard deviances (Std. Dev.) for all models used in this study.

| **Response variable** | **Model no** | **Bat : Condition** | | **Condition** | |
| --- | --- | --- | --- | --- | --- |
|  |  | **Var.** | **Std. Dev.** | **Var.** | **Std. Dev.** |
| **Maximum flight speed[m/s]** | **1** | 0.14 | 0.37 | 9.3 E-03 | 0.10 |
| **Maximum flight speed [m/s]** | **2** | 0.31 | 0.56 | 5.8 E-03 | 0.08 |
| **Total number of pulses** | **3** | 0.02 | 0.15 | 0.00 | 0.00 |
| **Number of multiple pulses** | **4** | 0.22 | 0.47 | 1.9 E-10 | 1.4 E-05 |
| **Number of doublets** | **5** | 0.08 | 0.28 | 1.2 E-09 | 3.5 E-05 |
| **Number of multiple pulses** | **6** | 0.19 | 0.44 | 9.1 E-09 | 9.6 E-05 |
| **Total number of pulses** | **7** | 4.8 E-03 | 0.07 | 9.4 E-10 | 3.1 E-05 |
| **\|𝚫pulse direction\|** | **8** | 0.00 | 0.00 | 0.00 | 0.00 |

**S10** Comparison between linear mixed effect models for maximum flight speed [m/s]. As described in Section *Statistical analysis*, the meandering width Δ*d* was significantly correlated with the flight number. Therefore, we modelled flight speed as a function of the flight number (model 1) as well as Δ*d* (model 2) in interaction with the acoustical condition and compared which of these models fitted the data better.

| **Model no** | **df** | **log(*Ը*)** | **AICc** | **Δ_i_** | ***w*_i_** | **R^2^_mar._ [%]** | **R^2^_cond._ [%]** |
| --- | --- | --- | --- | --- | --- | --- | --- |
| **1** | 7 | -18.913 | 57.4 | 0.0 | 1 | 33.0 | 71.6 |
| **2** | 7 | -30.614 | 80.8 | 23.4 | 0 | 33.5 | 88.1 |

Abbreviations: log(*Ը*) = log Likelihood, AICc = Akaike Information Criterion corrected for small sample sizes, Δ_i_ = difference in AICc values related to the best model, R^2^_mar._ = marginal pseudo R^2^ indicates the explained model variation without random effects, R^2^_cond._ = conditional pseudo R^2^ indicates the explained model variation including random effects

**S11** Summary lists the effect sizes (β), standard errors (SE), t-values (*t*) and z-values (*z*) of factor levels and their interactions from all generalised and linear mixed effect models used in this study, except model 6 (Supplemental Table S12).

| **Response variable** | **Model no** | **Variables**  **Statistics** | **Int** | **12** | **Perm** | **12 : Perm** | ***Δd*** | ***Δd* : Perm** | **S** | **S :**  **Perm** |
| --- | --- | --- | --- | --- | --- | --- | --- | --- | --- | --- |
| **Maximum flight speed[m/s]** | **1** | β | 2.55 | 0.28 | 0.08 | 0.62 | NA | NA | NA | NA |
|  |  | SE | 0.21 | 0.18 | 0.30 | 0.25 | NA | NA | NA | NA |
|  |  | *t* | 12.1 | 1.57 | 0.28 | 2.48 | NA | NA | NA | NA |
| **Maximum flight speed[m/s]** | **2** | β | 3.02 | NA | 1.79 | NA | 8.1 E-04 | 3.5 E-03 | NA | NA |
|  |  | SE | 0.35 | NA | 0.51 | NA | 6.4 E-04 | 9.1 E-04 | NA | NA |
|  |  | *t* | 8.58 | NA | 3.50 | NA | 1.27 | 3.81 | NA | NA |
| **Total number of pulses** | **3** | β | 3.93 | 0.32 | 0.03 | 0.26 | NA | NA | NA | NA |
|  |  | SE | 0.08 | 0.08 | 0.11 | 0.12 | NA | NA | NA | NA |
|  |  | *z* | 49.8 | 3.91 | 0.27 | 2.21 | NA | NA | NA | NA |
| **Number of multiple pulses** | **4** | β | 3.66 | 0.74 | 0.30 | 0.51 | NA | NA | NA | NA |
|  |  | SE | 0.19 | 0.10 | 0.27 | 0.17 | NA | NA | NA | NA |
|  |  | *z* | 19.5 | 7.17 | 1.12 | 2.94 | NA | NA | NA | NA |
| **Number of doublets** | **5** | β | 2.25 | 0.54 | 0.68 | 0.63 | NA | NA | NA | NA |
|  |  | SE | 0.16 | 0.15 | 0.21 | 0.19 | NA | NA | NA | NA |
|  |  | *z* | 13.9 | 3.60 | 3.23 | 3.25 | NA | NA | NA | NA |
| **Total number of pulses** | **7** | β | 4.88 | NA | 0.06 | NA | NA | NA | 0.41 | 0.02 |
|  |  | SE | 0.23 | NA | 0.31 | NA | NA | NA | 0.09 | 0.11 |
|  |  | *z* | 21.1 | NA | 0.19 | NA | NA | NA | 4.71 | 0.17 |
| **\|𝚫pulse direction\|** | **8** | β | 2.54 | 0.38 | 0.13 | 0.03 | NA | NA | NA | NA |
|  |  | SE | 0.11 | 0.17 | 0.16 | 0.24 | NA | NA | NA | NA |
|  |  | *z* | 24.0 | 2.27 | 0.85 | 0.12 | NA | NA | NA | NA |

Abbreviations: Int = Intercept, 12 = 12th flight, Perm = Permeable wall condition, *Δd* = meandering width, S = Maximum flight speed [m/s], NA = not available – these variables were not included in the respective model

**S12** Summary lists the effect sizes (β), standard errors (SE) and t-values (*t*) of factor levels and their interactions from the model of the number of multiple pulses as a function of a three-way-interaction between the flight number, the acoustic condition and the flight chamber section.

| **Response variable** | **Model no** | **Variables**  **Statistics** | Int | 12 | 2 | 3 | Perm | 12 : 2 | 12 : 3 | 12 : Perm | 2 : Perm | 3 : Perm | 12 : 2 : Perm | 12 : 3 : Perm |
| --- | --- | --- | --- | --- | --- | --- | --- | --- | --- | --- | --- | --- | --- | --- |
| ***Number of multiple pulses*** | **6** | β | 2.55 | 0.89 | 0.11 | 0.12 | 0.03 | 0.40 | 0.11 | 0.12 | 0.36 | 0.61 | 0.49 | 0.31 |
|  |  | SE | 0.20 | 0.19 | 0.14 | 0.15 | 0.28 | 0.25 | 0.28 | 0.27 | 0.20 | 0.23 | 0.38 | 0.47 |
|  |  | *t* | 13.0 | 4.70 | 0.78 | 0.82 | 0.10 | 1.63 | 0.38 | 0.44 | 1.76 | 2.68 | 1.29 | 0.67 |

Abbreviations: Int = Intercept, 12 = 12th flight, 2 = Flight chamber section 2 (Fig. 1*b*), 3 = Flight chamber section 3 (Fig. 1*b*), Perm = permeable wall condition; Interactions are indicated by colons
